# Single Mitochondrion Morphology‐Function Relationship Analysis Using Fluorescent Probes and Artificial Intelligence

**DOI:** 10.1002/advs.202509140

**Published:** 2025-08-21

**Authors:** Yang Ding, Bin Fang, Qingzhe Li, Biying Zhang, Jintao Li, Hua Bai, Nicolas H. Voelcker, Bo Peng, Xuekang Yang, Lin Li

**Affiliations:** ^1^ State Key Laboratory of Flexible Electronics (LoFE) & Institute of Flexible Electronics (IFE) Northwestern Polytechnical University Xi'an 710072 China; ^2^ State Key Laboratory of Flexible Electronics (LoFE) & Institute of Flexible Electronics (IFE Future Technologies) Xiamen University Xiamen 361102 China; ^3^ Department of Burns and Cutaneous Surgery Xijing Hospital The Fourth Military Medical University Xi'an 710072 China; ^4^ Drug Delivery Disposition and Dynamics Monash Institute of Pharmaceutical Sciences Monash University Parkville Victoria 3052 Australia

**Keywords:** artificial intelligence, fluorescent probes, image segmentation, mitochondria, single mitochondrion analysis

## Abstract

The ability to decode the relationship between mitochondrial morphology and function at the level of individual organelles is central to understanding cellular responses to stress, such as hypoxia. Herein, a comprehensive strategy is presented that integrates tailored fluorescent probes with artificial intelligence (AI) for single mitochondrion analysis. Focus is on three interrelated biomarkers, reactive oxygen species (ROS), viscosity, and mitochondrial membrane potential (MMP), that together form a pathophysiological axis indicative of mitochondrial state under hypoxic stress. A functional probe set is used to image these features simultaneously, including a newly developed dual‐cationic probe, **MitoVP**, which enhances mitochondrial targeting and resolution for viscosity sensing. Mitochondrial morphological features are then extracted using a deep learning‐based algorithm, which further classified individual mitochondria into dot, rod, and network morphotypes. This analysis enabled quantitative mapping between mitochondrial morphology and functional states, revealing significant heterogeneity across diverse physiological conditions. Based on this characterization, a random forest classifier trained on over 10,000 mitochondria accurately distinguished normoxic from hypoxic states and identified viscosity as a primary contributor to mitochondrial status under hypoxia. This integrated approach provides a powerful platform for single organelle investigations and advances the understanding of mitochondrial dysfunction in complex biological systems.

## Graphical abstract

An integrated strategy combining a mitochondria‐targeted fluorescent probe set and an AI framework enables simultaneous analysis of mitochondrial morphology and function at the single organelle level. By fusing multi‐parametric features, machine learning classification accurately distinguishes individual mitochondria under hypoxia‐related conditions.

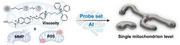

## 1 Introduction

Mitochondria are essential organelles that play central roles in diverse physiological and pathological processes.^[^ ^1^ ^]^ Their function and morphology are intimately linked, with functional impairments often accompanied by distinct structural hallmarks.^[^ ^2^ ^]^ For instance, in hypoxia‐related conditions such as myocardial infarction, ischemic stroke, and pulmonary hypertension, oxygen deprivation followed by reoxygenation imposes acute stress on mitochondrial homeostasis, resulting in irreversible damage to tissues and prolonged sequelae. Mechanically, hypoxia disrupts the electron transport chain, leading to excessive accumulation of reactive oxygen species (ROS), which in turn induces oxidative damage, increases mitochondrial matrix viscosity, and alters mitochondrial rheology. The resulting biophysical stress culminates in mitochondrial membrane potential (MMP) depolarization and cellular energy failure.^[^ ^3^, ^4^ ^]^ Morphologically, mitochondria show a transition from elongated, interconnected networks to fragment.^[^ ^5^ ^]^ Regarding mitochondrial function, ROS, viscosity, and MMP constitute an interrelated pathophysiological axis that reflects oxidative stress, microenvironmental rheology, and electrochemical integrity, offering a mechanistically coupled biomarker set essential for decoding mitochondrial states under hypoxic stress.^[^ ^6^ ^]^ Therefore, understanding these mitochondrial responses is pivotal for elucidating the pathological mechanisms of hypoxia‐driven diseases.

More importantly, within a single cell, mitochondria exhibit considerable heterogeneity in both morphology and function, influenced by their subcellular localization.^[^ ^7^ ^]^ This spatial variation enables compartmentalized metabolic regulation and localized signaling.^[^ ^8^ ^]^ Notably, cellular decisions such as apoptosis and differentiation are often initiated by a small population of dysfunctional mitochondria rather than a global collapse.^[^ ^9^, ^10^ ^]^ Therefore, characterizing behavior at the single mitochondrion level is critical for identifying early and spatially heterogeneous alterations in ROS, viscosity, and MMP, offering valuable insight into progression and therapeutic modulation of hypoxia‐related conditions.^[^ ^11^, ^12^, ^13^, ^14^ ^]^ However, key challenges, such as spatiotemporally accurate detection of mitochondrial biomarkers, hinder the application of single mitochondrion analysis in live‐cell.^[^ ^15^ ^]^ In mitochondrial viscosity measurements, some fluorescent probes exhibit limited targeting efficiency, leading to nonspecific cytosolic distribution and reduced imaging signal‐to‐noise ratio (SNR), which compromises the ability to acquire spatially and temporally aligned morphological and functional data.^[^ ^16^, ^17^ ^]^ Furthermore, the inherent complexity and dynamic variability of mitochondrial structures, coupled with their diverse network connectivity, pose significant challenges for morphological quantification.^[^ ^18^ ^]^ Changes in shape can directly affect the distribution and signal intensity of functional biomarkers, further complicating the precise assessment of form and function at the single mitochondrion level.^[^ ^19^ ^]^

Artificial intelligence (AI) has revolutionized microscopic image analysis, particularly through deep learning‐based segmentation methods that enable quantitative evaluation of complex subcellular structures.^[^ ^20^, ^21^ ^]^ Tools such as MitoSegNet and models by Sekh et al. have advanced the segmentation of mitochondrial images.^[^ ^22^, ^23^ ^]^ However, these methods remain limited in modeling the coordinated changes of morphology and function. We recently developed a deep learning‐based segmentation algorithm MoDL, which achieved state‐of‐the‐art performance in mitochondrial segmentation and elucidated correlations of morphology‐function at the global population level.^[^ ^24^ ^]^ However, this population‐level approach is inherently limited in resolving the heterogeneity and localized morphology‐function relationships present at the single mitochondrion level.

**Figure 1:**
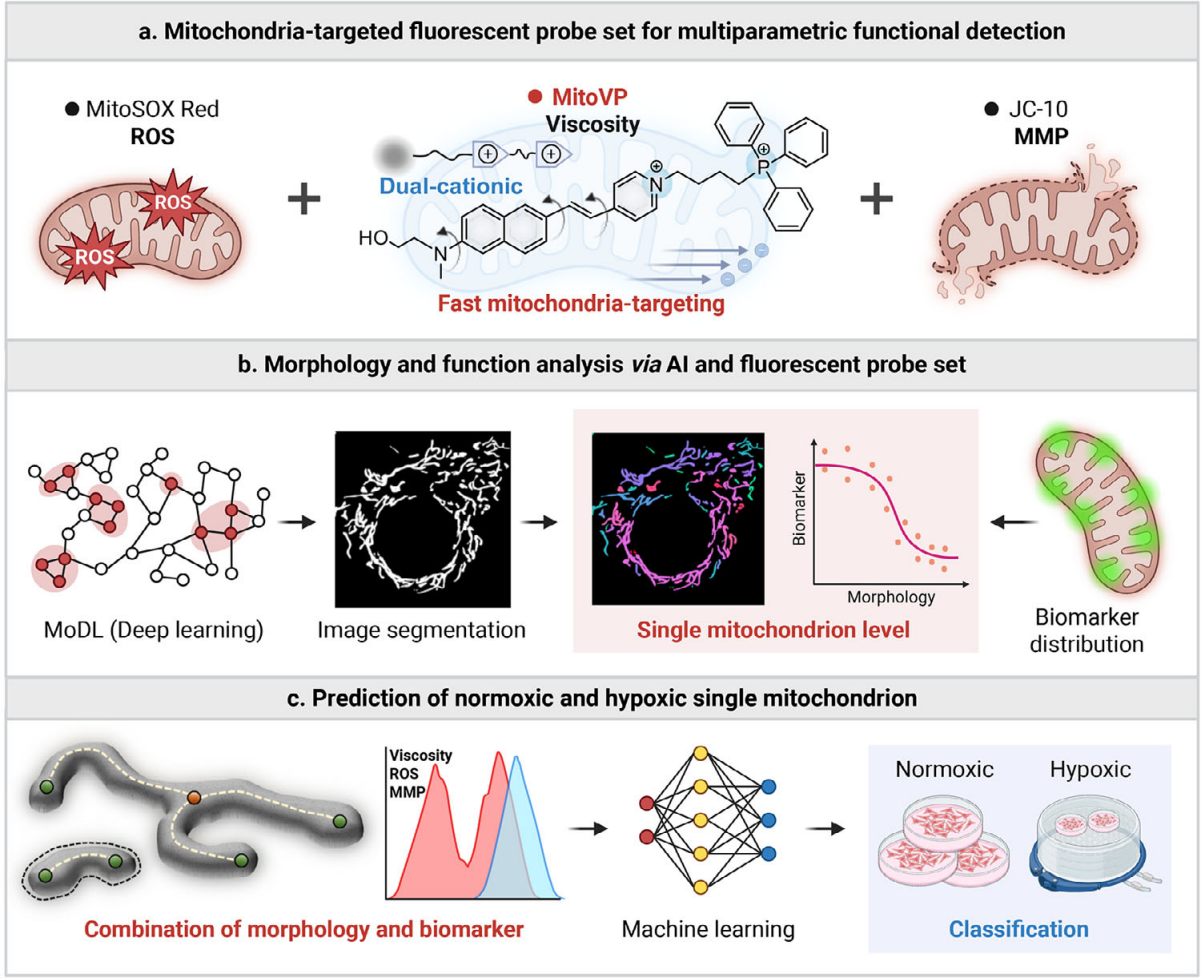
Schematic overview of the integrated workflow for investigating mitochondrial biomarker expression and morphology. a) Mitochondria‐targeted fluorescent probe set provides multiparametric functional assessment. The synthesized dual‐cationic fluorescent probe **MitoVP** detects mitochondrial viscosity, while the commercial MitoSOX Red and JC‐10 probes measure ROS and MMP, respectively. b) Deep learning‐based segmentation of mitochondrial images enables quantitative extraction of morphological features and correlation analysis with biomarker levels. c) Classification of normoxic and hypoxic mitochondria at the single organelle level using a machine learning algorithm trained on combined morphological and biomarker data.

Herein, we proposed a dual‐driven strategy that integrates a fluorescent probe set with AI to construct mappings between function and morphology at the single mitochondrion level. We specifically examined three key biomarkers: ROS, viscosity, and MMP, enabling multidimensional profiling of mitochondrial functionality under hypoxic stress. The fluorescent probe set includes a newly developed viscosity‐sensitive **MitoVP**, and two widely validated commercial probes for detecting ROS and MMP. To improve viscosity sensing, **MitoVP** was designed with a dual‐cationic structure to enhance mitochondrial targeting efficiency and imaging specificity, facilitating the simultaneous monitoring of mitochondrial morphology and function (**Figure** **1**a). Subsequently, we applied MoDL, a deep learning‐based algorithm, to segment individual mitochondria with high precision, enabling measurement of morphological parameters and spatial correlation with probe‐derived functional signals. To facilitate semantic context, each mitochondrion was classified into dot, rod, and network types, supporting biologically meaningful grouping for the investigation of structural and functional heterogeneity at the single mitochondrion level (Figure 1b). By merging these features, we trained a machine learning classifier on a dataset of 10,000 mitochondria to distinguish individual mitochondria under normoxic and hypoxic conditions (Figure 1c). Notably, feature importance revealed that mitochondrial viscosity is the most significant indicator, highlighting its critical role in reflecting functional state under hypoxia and the necessity of developing highly specific mitochondria‐targeted probes. Together, this synergistic strategy expands the analytical scope of single mitochondrion research, offering a novel paradigm for studying organelle heterogeneity and providing methodological insights relevant to early disease detection and biomarker discovery.

## 2 Results and Discussion

### 2.1 Structural Design and Photophysical Properties of MitoVP

**Figure 2:**
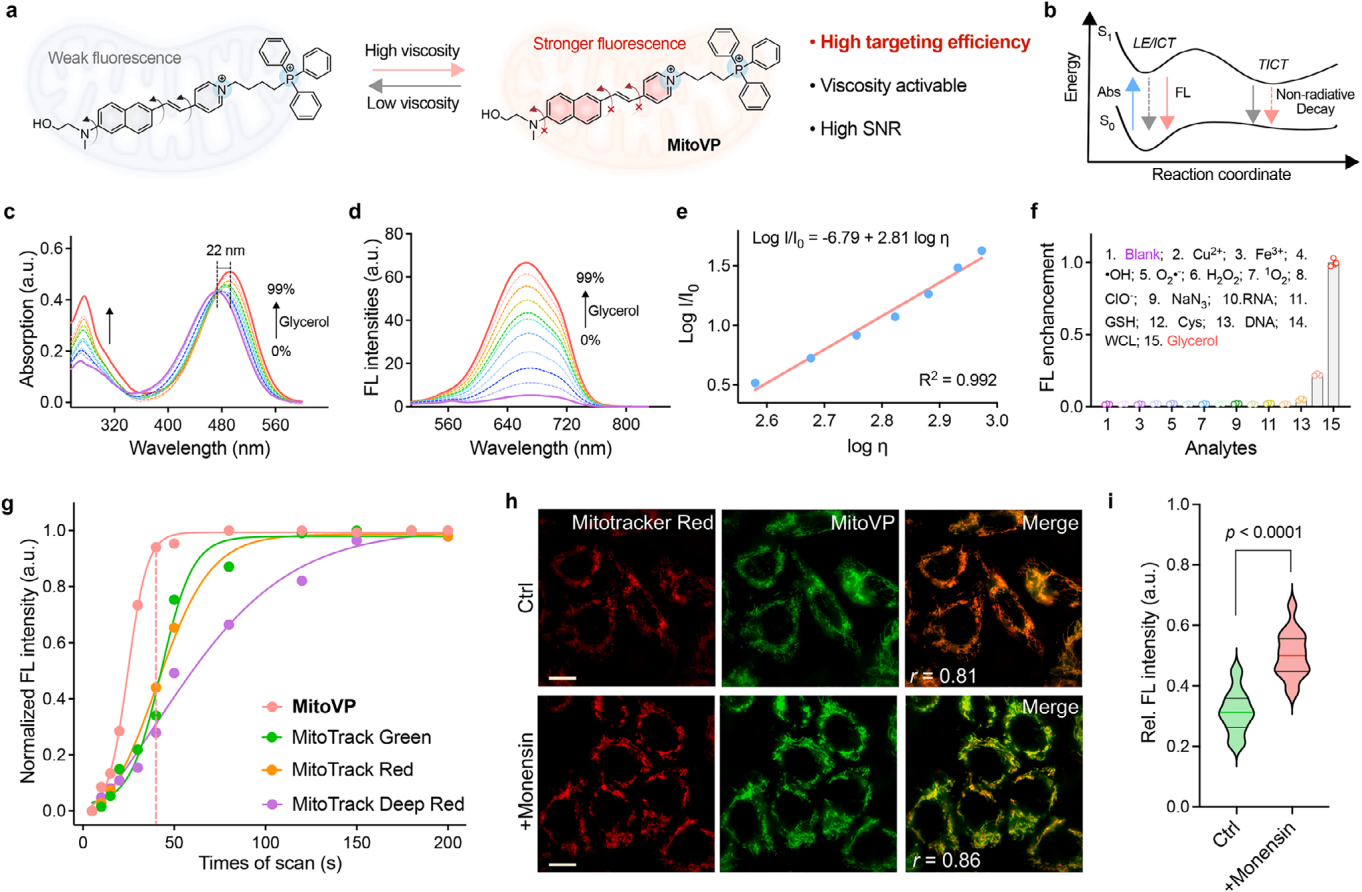
a) Molecular structure and sensing mechanism of the mitochondria‐targeted viscosity‐sensing probe **MitoVP**. b) Schematic diagram illustrating the energy potential surface based on the TICT effect for viscosity sensing. c) Changes of absorption and emission d) spectra of **MitoVP** (10 µm) after incubation with glycerol of different viscosities (0–99%). e) Linear relationship between log I_666_ and log η. f) Fluorescence response of **MitoVP** to interfering substances (100 µm), Cu^2+^, Fe^3+^, •OH, O_2_^•−^, H_2_O_2_, ^1^O_2_, ClO^−^, NaN_3_, RNA, GSH, Cys, DNA and WCL (Whole cell lysates), λ_ex_ = 492 nm; λ_em_ = 666 nm (viscosity), n = 3. g) Mitochondrial‐targeting efficiency of **MitoVP** and MitoTrackers in HepG2 cells. h) SIM images and Pearson's correlation coefficients (*r*) showing colocalization of MitoTracker Red (λ_ex_/λ_em_ = 579/599 nm, microscope channel: λ_ex_/λ_em_ = 561/605 nm, 500 nm, 30 min) and **MitoVP** (λ_ex_/λ_em_ = 480/666 nm, microscope channel: λ_ex_/λ_em_ = 488/700 nm, 2 µm, 30 min) in control (ctrl, *r* = 0.81) and monensin‐treated (+monensin, 10 µm, 30 min, *r* = 0.86) HepG2 cells, scale bar = 10 µm. i) Quantification of relative **MitoVP** fluorescence intensity in control and monensin‐treated HepG2 cells, n = 30, statistical differences were calculated using a two‐tailed Student's *t*‐test.

Mitochondrial viscosity probes often exhibit limited targeting efficiency, leading to prolonged cytoplasmic retention and increased nonspecific signals.^[^ ^25^, ^26^, ^27^ ^]^ To address these limitations, we designed **MitoVP**, featuring dual positively charged targeting groups: triphenylphosphine (TPP^+^) and a 4‐methylpyridine quaternary ammonium group. This dual‐cationic structure enhances targeting efficiency through stronger electrostatic interactions with the negatively charged mitochondrial membrane, promoting rapid accumulation, improved imaging specificity and SNR, thereby enhancing the precision of subsequent image analysis (**Figure** **2**a). Furthermore, **MitoVP** is designed to exhibit viscosity‐sensitive emission via a twisted intramolecular charge transfer (TICT) mechanism (Figure 2b). The pyridine and naphthyl groups are linked by a freely C = C bond, allowing unrestricted rotation that promotes fluorescence quenching in low‐viscosity. In contrast, increased viscosity restricts rotation, suppressing non‐radiative decay and enhancing fluorescence intensity. The structure of **MitoVP** was characterized in the Supporting Information.

Next, we investigated the viscosity‐sensitive ability and photophysical properties of **MitoVP**. As glycerol increased solution viscosity from 1.0 cP (water) to 950 cP (99% glycerol), the absorption spectra exhibited a red shift from 470 to 492 nm (Figure 2c). The emission intensity peak at 666 nm gradually increased, and the fluorescence quantum yield rose 36‐fold (QY_max_ = 0.54, Figures 2d and S1, Supporting Information). The linear relationship (R^2^ = 0.992) across a wide viscosity range, along with the low limit of detection (LOD, 0.83 cP), further confirms the probe's sensitivity (Figure 2e). Additionally, **MitoVP** exhibited minimal polarity dependence, with emission maxima varying slightly across solvents and displaying modest bathochromic shifts (Figure S2 and Table S1, Supporting Information). The probe's fluorescence intensity remained unaffected under simulated mitochondrial polarity conditions (20% DMSO),^[^ ^28^ ^]^ enabling viscosity sensitivity in cellular microenvironments (Figure S3, Supporting Information). Time‐resolved fluorescence analysis also confirmed the viscosity dependence, with lifetimes increasing from 0.21 ns (1 cP) to 1.42 ns (950 cP) due to restricted molecular motion (Figure S4, Supporting Information). Moreover, we assessed **MitoVP**’s selectivity toward various analytes and found that fluorescence activation at 666 nm occurred exclusively in viscous media, confirming its resistance to interference in biological systems (Figure 2f). **MitoVP** also demonstrated excellent stability across various pH and storage conditions (Figure S5, Supporting Information). Its amphiphilic structure further enhances cell‐permeability (logP_oct/wat_ = 0.06, lipophilicity, Figure S6, Supporting Information).^[^ ^29^ ^]^ The probe's aqueous solubility and photostability confirmed its suitability for live‐cell imaging (Figure S7, Supporting Information).

We also conducted time‐dependent density functional theory (TD‐DFT) and frontier molecular orbital (FMO) calculations. The optimized molecular revealed a large conjugated system, with the lowest unoccupied molecular orbital (LUMO) localized on the acceptor group and the highest occupied molecular orbital (HOMO) on the naphthalene group, consistent with TICT processes in the D‐π‐A structure. Upon excitation, the energy gap (ΔE_ST_) between the first excited state (S_1_) and the first triplet excited state (T_1_) was 1.18 eV, suggesting minimal ROS generation via intersystem crossing (ISC, Figure S8 and Table S2, Supporting Information). This property enhances photostability while minimizing phototoxicity during live‐cell imaging. Together, **MitoVP** exhibits excellent optical and viscosity‐sensing performance in vitro.

To further explore the potential of **MitoVP** in biological imaging, we assessed its cytotoxicity to confirm biocompatibility (Figure S9, Supporting Information). Subsequently, mitochondria‐targeting efficiency was validated in live‐cell imaging. Compared to commercial MitoTrackers, **MitoVP** exhibited superior membrane permeability and faster mitochondrial localization, revealing the effectiveness of the dual‐cationic structure in promoting targeting efficiency (Figure 2g). Furthermore, structured illumination microscopy (SIM) imaging demonstrated significant changes in mitochondrial fluorescence intensity following treatment with monensin (an ionophore to increase viscosity, Figure 2h,i). The clear visualization and colocalization coefficients (*r* > 0.8) with MitoTracker were observed in **MitoVP** (2 µm), confirming its optimal working concentration and excellent targeting capability (Figure S10, Supporting Information). Together, the enhanced imaging properties of **MitoVP** provide a robust foundation for precise analysis of the relationship between mitochondrial viscosity and morphology.

### 2.2 Morphology‐Function Relationship Analysis at the Single Mitochondrion Level

**Figure 3:**
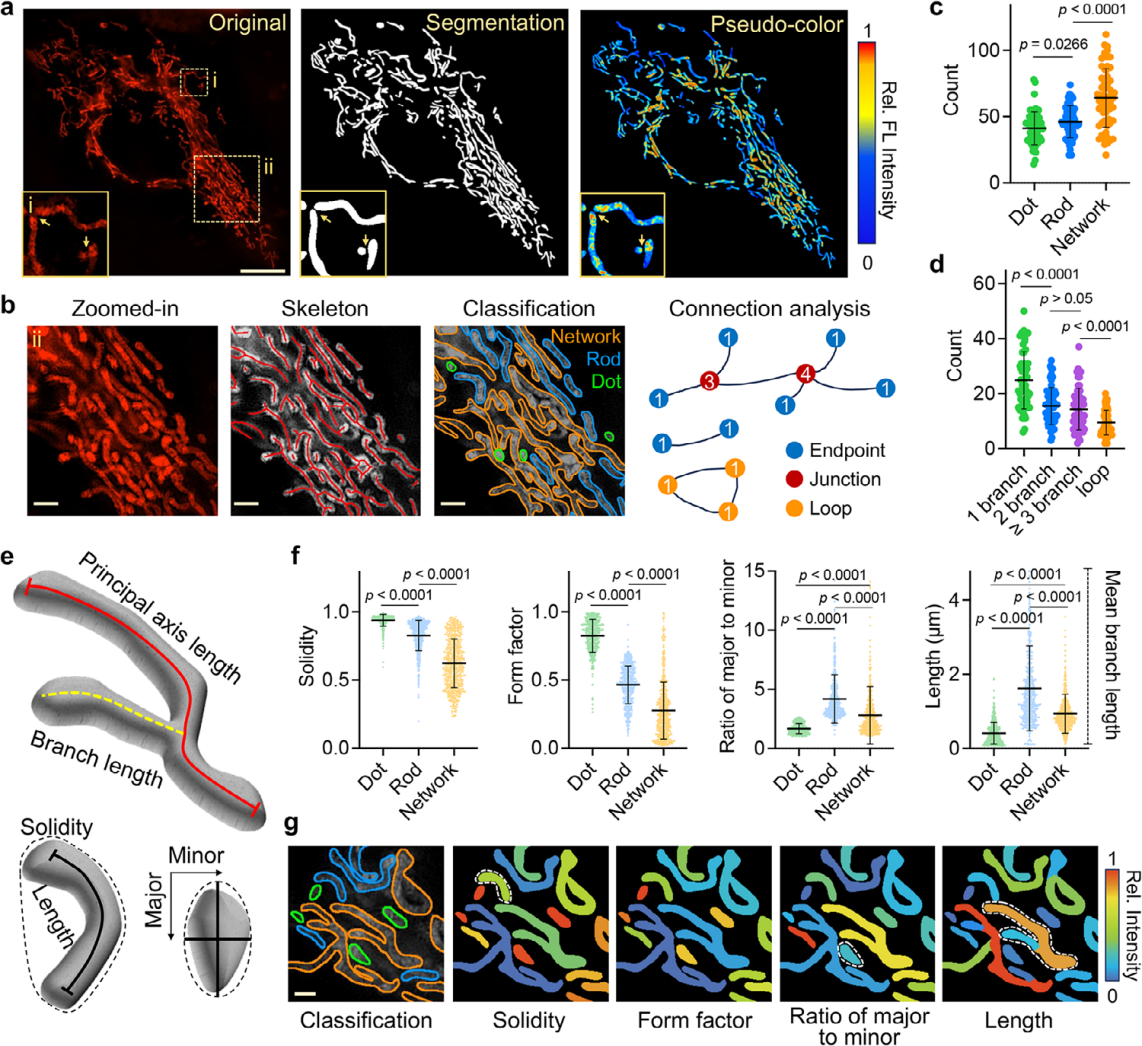
Image segmentation and quantitative analysis of mitochondrial morphology in HepG2 cells imaging. a) Raw SIM image, segmentation mask, and pseudo‐color map obtained by deep learning‐based MoDL algorithm, scale bar = 10 µm. The magnified inset (i) demonstrates accurate segmentation of closely apposed mitochondria. b) Region (ii) presents a skeletonized representation used for morphological classification of mitochondrial structure, distinguishing network (orange), rod (blue), and dot (green) types, followed by connectivity analysis of endpoints, junctions, and loops, scale bar = 2 µm. c) Quantification analysis of mitochondrial classified into dot‐, rod‐, and network‐shaped structures, n = 60 images. d) Distribution of mitochondria with varying numbers of branches and loops, n = 60 images. e) Schematic illustration of key mitochondrial parameters, including branch information, solidity, and ratio of major to minor. f) Comparison of solidity, form factor, ratio of major to minor axis, and length across the dot, rod, and network morphological types, n = 578 mitochondria. g) Representative mapping of these shape features onto the segmentation mask, scale bar = 1 µm. Statistical differences were calculated using a two‐tailed Student's *t*‐test (c, d, and f).

Previously, we developed a deep learning‐based MoDL algorithm to establish morphology‐function correlations by analyzing the mitochondrial population as a whole within each imaging field (**Figure** **3**a).^[^ ^24^ ^]^ However, because the functional data were collected from biochemical assays performed at the whole‐cell level, the approach was limited to population‐averaged measurements and was unable to resolve morphology‐function relationships at the single mitochondrion level. Herein, we extended this framework to the single mitochondrion level by integrating a functional probe set with MoDL. To provide semantic interpretation of raw mitochondrial morphometric data, we expanded the MoDL pipeline with a morphological type of analysis module that map individual mitochondrial function and morphology information. By examining a zoomed‐in region, skeletonization was applied to segmented masks. Based on extracted features, each mitochondrion was categorized into dot, rod, and network types, enabling biologically meaningful grouping for downstream morphology‐function analyses. Furthermore, node‐based connectivity analysis was performed to quantify network topology, including endpoints, junctions, and loops. This revealed diverse branching characteristics across mitochondrial subtypes (Figure 3b). Quantitative analysis of morphological type distribution in living cells indicated that networks were the most prevalent, often characterized by one or two branches (Figure 3c,d).

To validate the biological relevance of morphological type, we quantitatively analyzed morphological parameters of single mitochondrion across three categories (Figure 3e). The results indicate that dot mitochondria exhibit higher solidity and form factor, consistent with their uniform and compact appearance. Rod and network mitochondria showed a higher major‐to‐minor axis ratio, reflecting their elongated geometry. Moreover, network mitochondria display broader standard deviation distributions, indicating greater structural complexity and diverse interconnectivity. Despite their intricate branching, network mitochondria had shorter mean branch length compared to the rod category, highlighting the extended principal axis characteristic of rod‐shaped mitochondria (Figure 3f). Mapping these measurements onto segmentation masks establishes a direct visual correlation between quantitative indices and structural features (Figure 3g). Collectively, these findings demonstrate the effectiveness of deep learning‐based segmentation in live‐cell imaging and highlight its potential for single mitochondrion studies across a wide range of applications.

Next, to investigate the relationship between morphology and functional biomarkers at the single mitochondrion level, we combined MoDL with a mitochondria‐targeted fluorescent probe set comprising **MitoVP** for viscosity sensing, and two widely validated commercial probes, MitoSOX Red and JC‐10, for ROS and MMP detection. This probe set provides an integrated and comprehensive view of redox imbalance, structural remodeling, and energy failure, forming a mechanistically coherent indicator set for characterizing mitochondrial dysfunction. Mechanically, disrupted electron transport under hypoxia leads to excess ROS, which induces oxidative damage to membrane lipids and proteins, increases matrix viscosity, and alters mitochondrial microstructure. These physical changes impair membrane dynamics and ion transport, ultimately causing MMP depolarization and metabolic collapse.^[^ ^3^, ^4^, ^5^, ^6^ ^]^ This multidimensional biomarker profiling enables characterization of structural and functional heterogeneity at the single mitochondrion level.

**Figure 4:**
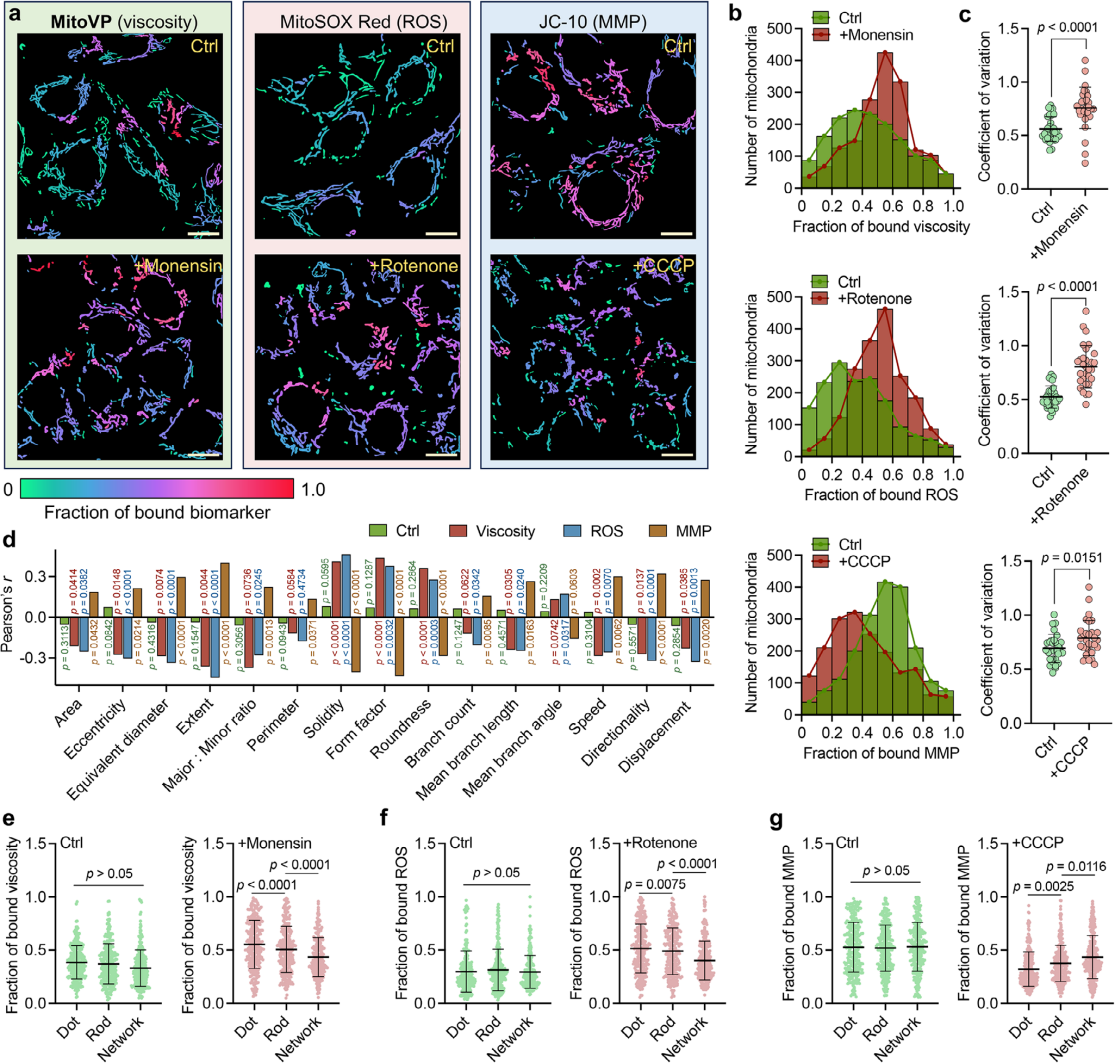
Assessment of mitochondrial biomarkers and morphological features under different treatments. a) Representative pseudo‐color maps illustrating mitochondrial biomarker intensities, including viscosity, ROS, and MMP, obtained by multiplying the segmented masks with raw fluorescence images. HepG2 cells were treated with monensin (10 µm, 1 h), rotenone (10 µm, 4 h), or CCCP (10 µm, 4 h), followed by staining with fluorescent probe set: **MitoVP** (2 µm, 30 min), MitoSOX Red (5 µm, 30 min), JC‐10 (10 µm, 30 min) was used to measure mitochondrial viscosity, ROS level, and MMP depolarization, respectively, scale bar = 10 µm. b) Histograms depicting the distribution of mitochondrial fraction of bound viscosity, ROS, and MMP in control and drug‐treated cells. c) Coefficients of variation comparing control and drug‐treated groups. d) Pearson's correlation coefficients (*r*) between mitochondrial morphological parameters and the fraction of bound viscosity, ROS, and MMP. e) Comparison of fraction of bound viscosity, ROS, and MMP among mitochondria classified as dot‐, rod‐, or network‐shaped structures in control and monensin‐, rotenone‐ f), and CCCP‐treated g) HepG2 cells, n = 274 mitochondria. Statistical differences were calculated using a two‐tailed Student's *t*‐test (c, e, f, and g).

Subsequently, each biomarker level (i.e., mitochondrial viscosity, ROS levels, and MMP depolarization) was selectively modulated via the addition of monensin, rotenone, and carbonyl cyanide 3‐chlorophenylhydrazone (CCCP, an oxidative phosphorylation uncoupler), respectively. To visualize the spatial distribution of biomarker signals, raw fluorescent intensities were overlaid onto the segmented masks, producing pseudo‐color functional maps representing the bound fraction of each biomarker (**Figure** **4**a). We observed alterations in overall mitochondrial morphology, characterized by an increased prevalence of dot‐ and rod‐shaped structures and a reduction in network formation under following each drug treatment (Figure S12, Supporting Information). Meanwhile, control cells exhibited a relatively homogeneous biomarker distribution, whereas drug treatment altered the bound fraction of viscosity, ROS, and MMP (Figure S13, Supporting Information). The histograms showed that the distributions of biomarker fractions followed a normal pattern, but that monensin and rotenone treatments shifting viscosity and ROS fractions toward higher values, while CCCP‐treated mitochondria exhibited a reduction in MMP fraction (Figure 4b). We also found that drug‐treated cells exhibited higher variation in their biomarker fractions, suggesting increased functional heterogeneity among individual mitochondria (Figure 4c). Time‐lapse imaging revealed changes in dynamic behavior at the single mitochondrion level following each drug treatment, including reduced motility speed, loss of directional persistence, and shortened displacement trajectories (Figures S14,S15, Supporting Information). These findings highlight that mitochondrial dysfunction disrupts energy‐dependent trafficking and structural coordination, leading to impaired motility and spatial organization.

Correlation analysis at the single mitochondrion level revealed that viscosity exhibited a positive with roundness and a negative with the ratio of major to minor and extent. This suggests that an elevated viscosity fraction corresponds to a less elongated form, potentially reflecting membrane or matrix alteration that leads to a swollen phenotype. ROS and MMP fraction correlated positively with solidity and form factor but negatively with mean branch length, indicating a transition from a networked configuration to fragmented mitochondria, characteristic of a fission‐dominant state. Furthermore, mitochondrial motility speed, directionality, and displacement showed positive correlation with viscosity and ROS, and a negative with MMP, suggesting that impaired dynamic activity is closely linked to mitochondrial dysfunction. Moreover, mean branch angle exhibited minimal correlation with any biomarker, suggesting that angle‐based parameters alone may be insufficient to distinguish functional variations (Figure 4d).

While these morphological parameters analysis provide detailed descriptors, they also lack intuitive biological meaning. To further enhance semantic clarity, we compared biomarker fraction across the defined three morphological types. Under control conditions, all categories displayed uniform biomarker distributions. However, following treatment with monensin or rotenone, dot‐shaped mitochondria exhibited significantly higher viscosity and ROS fractions than rods and networks. Conversely, CCCP treatment resulted in higher MMP in network mitochondria and lower levels in dots. These observations emphasize that distinct morphological types exhibit differential sensibility to dysfunction, which may be attributed to their inherent structural properties, such as interconnectivity, fusion/fission capacity, or buffering potential (Figure 4e‐g).^[^ ^30^, ^31^ ^]^ Together, these findings reveal the necessity of single mitochondrion analysis for capture functional and structural diversity in response to physiological conditions.

### 2.3 Hypoxic Stress Classification at the Single Mitochondrion Level

**Figure 5:**
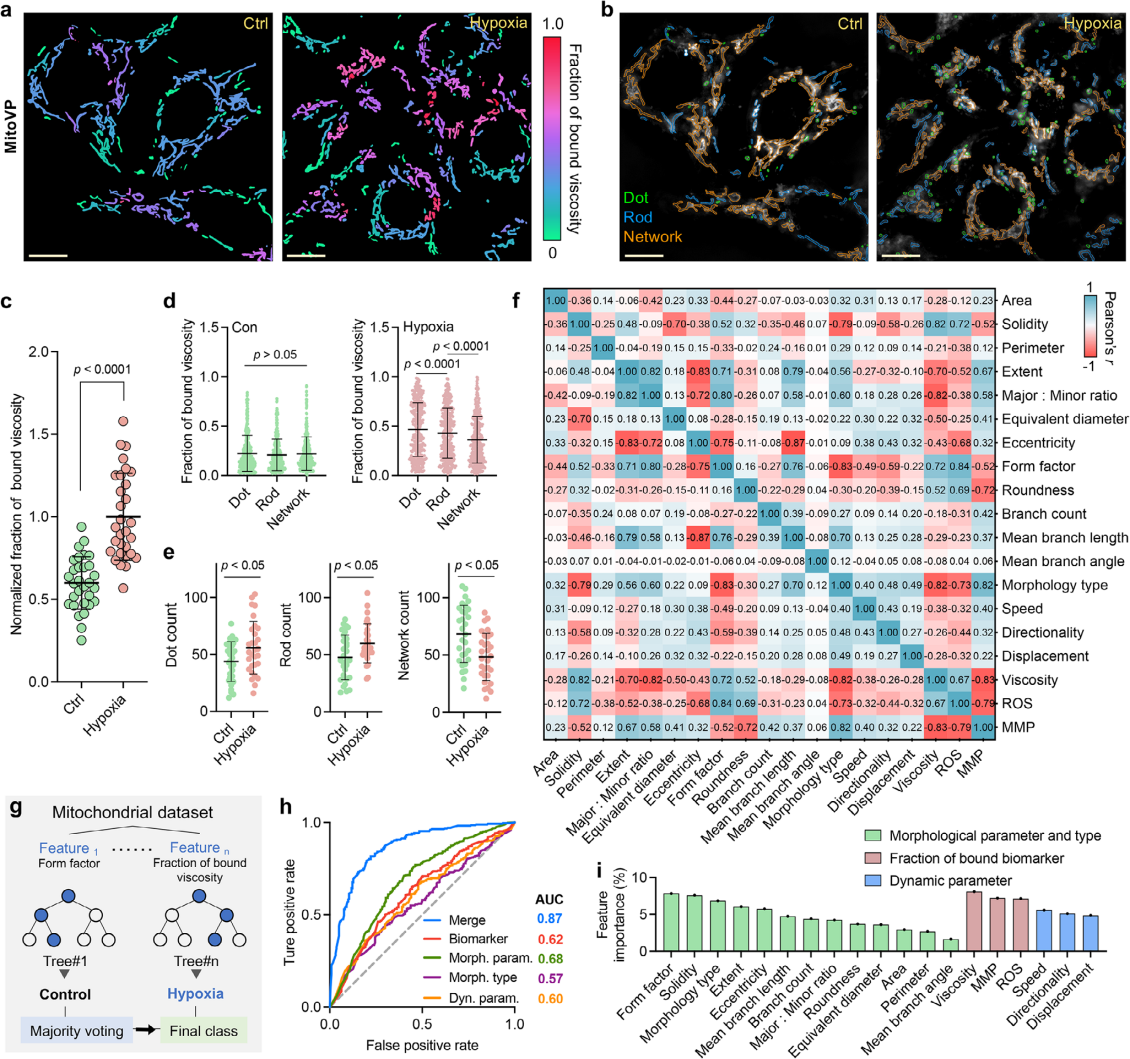
Classification of normoxic (control) and hypoxic mitochondria using machine learning. a) Representative merged pseudo‐color and corresponding shape‐classified images b) in control and hypoxia‐treated HepG2 cells. Hypoxic condition was performed by culturing cells under 1% O_2_ for 6 h, followed by staining with **MitoVP** (2 µm, 30 min) prior to imaging, scale bar = 10 µm. c) Normalized fraction of bound viscosity in control and hypoxic HepG2 cells, n = 30 images. d) Comparison of the fraction of bound viscosity among mitochondria classified as dot, rod, or network under control and hypoxic conditions, n = 429 mitochondria. e) Quantification of mitochondria categorized into dot, rod, and network in control and hypoxic cells, n = 30 images. f) Correlation matrix illustrating interrelationships among mitochondrial biomarkers, morphological parameters, and type labels. g) Schematic representation of the random forest model (a machine learning algorithm that aggregates multiple decision trees) trained on 10000 mitochondria to distinguish between control and hypoxic conditions. The classifier's final decision was determined by majority voting. h) ROC curves and AUC values evaluating the performance of the random forest classifier using various feature sets. (i) Feature importance identifying the relative contributions of morphological and biomarker features for mitochondrial classification under control and hypoxic conditions. Statistical differences were calculated using a two‐tailed Student's *t*‐test (c, d, and e).

Building on this integrated profiling system, we lastly applied it to examine how hypoxia‐driven stress influences mitochondrial morphology and function. Hypoxia impairs mitochondrial function by reducing oxygen availability, influencing ROS levels, viscosity, and MMP depolarization, which collectively contribute to structural remodeling and physiological disruption.^[^ ^32^, ^33^ ^]^ To investigate these changes at the single mitochondrion level, we established a hypoxia model and employed a probe set in combination with MoDL to quantify morphological and functional alterations. The merged pseudo‐color and shaped‐classified images simultaneously visualize biomarker signal distribution and morphology types (**Figures** **5**a,b and S16, Supporting Information). The results showed a significant alternation in mitochondrial function under hypoxia, particularly pronounced in dot‐shaped structures, highlighting the spatial correspondence between biomarker distribution and morphological phenotype at the single mitochondrion level (Figures 5c,d and S17, Supporting Information). Meanwhile, morphological analysis showed a notable increase in dot‐ and rod‐shaped mitochondria, coupled with a significant decrease in interconnected network structures in hypoxic cells (Figures 5e and S18, Supporting Information). This morphological shift suggests diminished mitochondrial complexity and enhanced fragmentation, a state typically linked to impaired mitochondrial function and energy depletion. Moreover, time‐lapse imaging and dynamic analysis demonstrated a suppression of mitochondrial motility capacity under hypoxia, consistent with an adaptive stress response to oxygen deprivation and metabolic disorder (Figures S19,S20, Supporting Information).

The description of the morphology, dynamics, and biomarker mapping of individual mitochondria at the single mitochondrion level confirms the significance of mitochondrial biomarker imaging (Figure 5f), further encouraging us to achieve more precise functional predictions at the macroscopic level. Subsequently, we constructed a random forest classifier based on an integrated feature set encompassing biomarker data, morphology parameters, and type labels, and dynamic parameters (Figure 5g). A dataset comprising 10,000 mitochondria was assembled and randomly split (7: 3) into training and test sets. Furthermore, the classifier's performance was evaluated using receiver operating characteristic (ROC) and area under the curve (AUC), which highlighted the relative contributions of each feature set. When only biomarker fractions, dynamic parameters, or morphology type labels were used, the AUC remained modest, presumably due to limited feature diversity. Morphological parameters, while more diverse, offered only moderate improvement, possibly due to their relatively low correlation. Notably, integration of all feature sets significantly enhanced model performance, achieving the highest classification accuracy (AUC = 0.87, Figure 5h and Table S5, Supporting Information). This result was further validated by 5‐fold cross‐validation and repeated training with different random seeds (Table S6, Supporting Information). Additionally, we extended the classifier to additional cell types and retrained the model. The results showed comparably high AUC values (L02 = 0.89, HeLa = 0.86), supporting the model's applicability across diverse cell lines (Figure S21, Supporting Information). These findings demonstrate the complementary roles of structural and functional features in single mitochondrion classification.

Feature importance quantifies the relative impact of each variable on classification decisions. The results indicate that form factors (7.8%), solidity (7.6%), and extent (6.0%) are among the top contributors within the morphological parameter set, suggesting that hypoxia‐driven mitochondrial remodeling is characterized by a transition from continuous structures to smaller, rounder morphologies that occupy a lower proportion of their bounding space and have lower overall integrity. Morphology types (6.8%) also showed a high contribution, highlighting their value as a compact and semantically meaningful representation of structural organization. Among the biomarker metrics, the fraction of bound viscosity (8.1%) proved particularly influential, potentially because viscosity is highly sensitive to alterations in protein/lipid composition, matrix density, and membrane organization, all of which can be directly affected by oxygen deprivation (Figure 5i).^[^ ^34^ ^]^

To further explore mitochondrial heterogeneity within each condition, we performed unsupervised clustering of individual mitochondria using the K‐means (*k* = 2).^[^ ^35^ ^]^ Principal component analysis (PCA) visualization revealed a separation into distinct subpopulations under both control (silhouette score = 0.22) and hypoxic stress (silhouette score = 0.25, Figure S22, Supporting Information). Additionally, several features exhibited significant differences between the two subclusters (*p* < 0.05), suggesting the presence of intrinsic heterogeneity (Table S7, Supporting Information). Furthermore, clustering assignment probabilities were quantified and incorporated as an additional feature into the random forest classifier. This led to a moderate improvement in predictive performance (AUC = 0.88), indicating the potential value of mitochondrion heterogeneity in contributing classification robustness.

Considering that hypoxia is a continuous biological process,^[^ ^4^ ^]^ we extended the classifier to a multi‐label model to represent varying degrees of hypoxia. Based on cell viability across different hypoxia durations, labels were defined as control, mild, moderate, and severe hypoxia (Figure S23, Supporting Information). Quantitative analysis revealed a progressive increase in viscosity and ROS fractions, accompanied by a corresponding decrease in MMP levels. These findings confirm that distinct hypoxia levels induce graded mitochondrial dysfunction. By integrating all feature sets, the multi‐label prediction demonstrated robust performance (Macro‐AUC = 0.80). Feature importance analysis identified solidity (7.8%), extent (7.3%), and ROS fraction (7.2%) as the top three contributors, highlighting their significance in characterizing mitochondrial responses across a continuum of hypoxic stress (Figure S24, Supporting Information).

## 3 Conclusion

In summary, we developed an integrated strategy that combines a fluorescent probe set with AI technology to explore the relationship between function and morphology at the single mitochondrion level. The probe set specifically examines three interrelated biomarkers: ROS, viscosity, and MMP, which collectively provide a complementary mitochondrial physiological profile under stress conditions. For viscosity detection, **MitoVP** was designed with a dual‐cationic structure to address the insufficient targeting efficiency of existing viscosity probes, demonstrating superior specificity and improved imaging SNR. Additionally, a deep learning‐based image segmentation model was employed for accurate mitochondrial identification and morphology feature extraction, which further classified individual mitochondria into dot, rod, and network morphotypes. This analysis enabled quantitative mapping between mitochondrial morphology and functional states in single mitochondrion level, revealing significant heterogeneity across diverse physiological conditions. By merging biomarker data, morphological parameters and type labels, and dynamic parameters, we trained a machine learning classifier that accurately predicted normoxic or hypoxic states at the single mitochondrion level, highlighting its potential for hypoxia‐related disorder such as myocardial infarction and ischemic stroke. Notably, feature importance scores identified viscosity as a top contributor, suggesting its relevance under hypoxic stress and highlighting the necessity for precision‐targeted mitochondrial viscosity probes. Looking forward, this strategy holds potential for expansion to multiplexed sensing integrated with morphology analysis^[^ ^36^ ^]^ providing comprehensive insights into mitochondrial studies, opening exciting avenues for single mitochondrion level investigations in live‐cell biological research.

## 4 Experimental Section

### Fluorescence Quantum Yields

The fluorescence quantum yields (Φ) were determined by using rhodamine B (RhB) as the reference.^[^ ^37^ ^]^ Absorption and fluorescence spectra of **MitoVP** and RhB were measured under identical conditions.

### Limit of Detection

The LOD was determined using the standard method.

(1) $$\mathrm{LOD}=\frac{3\sigma }{k}$$

where *σ* is the standard deviation of blank measurements (0% glycerol, n = 10), and k is the slope of the linear calibration curve between fluorescence intensity at 666 nm and log(η). The calculated LOD corresponds to a viscosity change of ≈0.83 cP, indicating the probe's ability to detect subtle increases from aqueous baseline conditions.

### TD‐DFT Calculations

All calculations were conducted in the Gaussian 09 program. Ground‐state geometry optimization was carried out with B3LYP hybrid functional. Time‐dependent density functional theory (TD‐DFT) was subsequently applied to compute the excited‐state properties. The geometry optimization of singlet‐singlet excitation energies was carried out with a basic set composed of 6–31G(d) for carbon (C), nitrogen (N), sulfur (S), and hydrogen (H) atoms. The lowest 25 spin‐allowed singlet‐singlet transitions, up to an energy of ≈5 eV, were considered in the absorption spectra calculations.^[^ ^38^ ^]^

### Cell Viability Assay

Cell viability was analyzed using PrestoBlue cell viability reagent (Invitrogen, Carlsbad, CA). PrestoBlue was diluted 10‐fold in fresh medium and directly added to the cells. After incubation at 37 °C for 1 h, absorbance was read at 570 nm using a microplate reader. Data was expressed as a percentage of untreated control cells. Higher absorbance values indicate greater overall metabolic activity.

### Cell Culture and SIM Super‐Resolution Imaging

HepG2 cells (human hepatocarcinoma cells) were obtained from American Type Culture Collection (ATCC). Cells were cultured under standard conditions (95% humidity, 5% CO_2_, 37 °C) and harvested by 0.5% (w/v) trypsin‐EDTA solution. Cells were seeded in confocal dishes to ≈60% confluence before treatment. Cells were then treated according to the experimental group requirements and subsequently incubated with **MitoVP** (2 µm, 30 min), MitoSOX (5 µm, 30 min), or JC‐10 (10 µm, 30 min) at 37 °C. After staining, cells were rinsed with PBS and imaged using a Nikon N‐SIM E microscope equipped with a CFI Apochromat TIRF 100XC Oil (N.A. ≥1.49, WD ≥ 0.12 mm). The co‐localization experiments were performed with a dual‐channel mode, and the Pearson's correlation coefficient (*r*) was calculated using Cell Profiler with the co‐localization module.

### Targeted Mitochondrial Viscosity Analysis

In SIM mode, a cell field of view was selected as the observation area. Following a meticulously designed experimental procedure, multiple fluorescent probes were sequentially introduced into the imaging culture dish, and immediately initiating SIM for continuous imaging after each addition. The time interval set to 1 s and the exposure time to 10 ms to ensure high temporal resolution. Imaging continues until the mitochondrial reached a stable state of fluorescence intensity, where the signal saturates and remains relatively unchanged over time.

### Pseudo‐color Image Generation

The pseudo‐color image generation method was implemented in Python. First, each raw image was converted to an 8‐bit grayscale format. A corresponding binary segmentation mask was then generated by thresholding (with pixels >127 set as foreground). Distinct connected regions were extracted from the binary mask using connected‐component labeling. For each labeled region, the average grayscale intensity was computed. This intensity value, ranging from 0 to 255, was subsequently mapped to a pseudo‐color using continuous linear interpolation between predefined color anchors. The computed pseudo‐color was assigned to the pixels within each region, and the resulting pseudo‐colored images were saved for further analysis.

### Skeletonization and Mitochondrial Classification

Raw images and corresponding binary segmentation masks were acquired with consistent dimensions, which were then processed using a thinning algorithm to create one‐pixel‐wide skeletons. To improve visualization, the skeletons were dilated using a 3 × 3 kernel and superimposed on the raw images in red. Subsequently, connected regions were identified through connected‐component labeling, and morphological properties such as major and minor axis lengths, solidity, and from factor were measured for each region. Branch point count was computed on the skeletonized mask using a defined neighborhood approach. Based on the branch count and morphological features, each mitochondrial structure was classified into one of three categories: network, rod, or dot. Finally, the contours of these regions were outlined with distinct colors corresponding to their respective classification.

### Morphological Semantic Classification

The semantic classification criteria for dot‐, rod‐, and network‐shaped mitochondria were based on a combination of mitochondrial skeleton branching and geometric features. Dot‐shaped mitochondria were defined as structures with no branching (Branch count = 0), a low elongation ratio (Ratio of major to minor < 2), and a relatively round shape (Roundness > 0.25). Rod‐shaped mitochondria were also unbranched (Branch count = 0), but exhibit an elongated morphology (Ratio of major to minor ≥ 2) and lower roundness (Roundness ≤ 0.25). Network‐shaped mitochondria were characterized by at least one branch point (Branch Count ≥ 1), forming either tree‐like or closed‐loop structures.

### Statistical Analysis

The experiment was independently repeated and depicted in figure legends. All representative images presented were reproduced with similar results. The experimental data were analyzed with GraphPad Prism 9 (GraphPad Software, San Diego, CA) and presented as mean ± standard deviation (s.d.). The experiments were independently repeated at least 3 times. Normality and homogeneity of variances were assessed using the Shapiro‐Wilk test and Brown Forsythe test, respectively. Statistical comparisons were conducted using one‐way ANOVA followed by Dunnett's multiple comparison test (more than two groups) or two‐tailed Student's *t*‐test (two groups). A *p* value (<0.05) was considered statistically significant.

## Conflict of Interest

The authors declare no conflict of interest.

## Supporting information



Supporting Information

## Data Availability

The data that support the findings of this study are available from the corresponding author upon reasonable request.
