# Supplementary material for: Single Mitochondrion Morphology‐Function Relationship Analysis Using Fluorescent Probes and Artificial Intelligence
Source: Adv Sci (Weinh). 2025 Aug 21;12(43):e09140. doi: 10.1002/advs.202509140 (PMC12631887; doi:10.1002/advs.202509140)
Supplement: Supplementary file 1 — Supporting Information [file ADVS-12-e09140-s001.pdf]

Supporting Information

Single Mitochondrion Morphology-Function Relationship Analysis Using Fluorescent Probes and Artificial Intelligence

Yang Ding,[a]# Bin Fang,[b]# Qingzhe Li,[c]# Biying Zhang,[a] Jintao Li,[a] Hua Bai,[a] Nicolas H. Voelcker,[d] Bo Peng,[a]* Xuekang Yang,[c]* and Lin Li[a],[b]*

[a] Y. Ding, B. Zhang, J. Li, Prof. Dr. H. Bai, Prof. Dr. B. Peng, Prof. Dr. L. Li
State Key Laboratory of Flexible Electronics (LoFE) & Institute of Flexible Electronics (IFE)
Northwestern Polytechnical University
Xi'an 710072, China
*Corresponding author(s): Prof. Dr. B. Peng (iambpeng@nwpu.edu.cn)

[b] Dr. B. Fang, Prof. Dr. L. Li
State Key Laboratory of Flexible Electronics (LoFE) & Institute of Flexible Electronics (IFE, Future Technologies)
Xiamen University
Xiamen 361102, China
*Corresponding author(s): Prof. Dr. L. Li (ifelli@xmu.edu.cn)

[c] Q. Li, Prof. Dr. X. Yang
Department of Burns and Cutaneous Surgery, Xijing Hospital
The Fourth Military Medical University
Xi'an 710072, China
*Corresponding author(s): Prof. Dr. X. Yang (sskzzb06@fmmu.edu.cn)

[d] Prof. Dr. N. H. Voelcker
Drug Delivery, Disposition and Dynamics, Monash Institute of Pharmaceutical Sciences
Monash University
Parkville, Victoria 3052, Australia

[#] These authors contributed equally to this work

Table of Contents

1. **Reagents and Instruments** 2
2. **Chemical Synthesis** 3
3. **Results and Discussion** 5
   3.1 Supplemental Figures 5
   3.2 Supplemental Tables 18
4. **Characterization Spectra** 21
5. **Commands for Theoretical Calculation of MitoVP** 24
6. **References** 26

1. Reagents and Instruments

Unless otherwise noted, all chemicals were purchased from commercial suppliers and used without further purification. All reactions were performed under dry nitrogen protection. Reaction progress was monitored by TLC on pre-coated silica plates, with spots being visualized by UV light activation or iodine staining. Silica gel 60 (200-300 mesh, Silicycle) was used for column chromatography. *N*,*N*-dimethylformamide (DMF) and dichloromethane (DCM) were distilled over CaH_2. Petroleum ether (PE, 60-90ºC), ethyl acetate (EA) and methanol (MeOH) were used as eluents for flash column chromatography with Merck silica gel (0.040-0.063 mm). Piperidine, 4-(bromomethyl)benzeneboronic acid pinacol ester and 4-(dimethylamino)benzaldehyde were purchased from Aladdin. Cysteine (Cys), glutathione (GSH), H_2O_2, NaClO, and other reagents were used as received from Sigma and Shanghai Chemicals Ltd. MitoTrackers, MitoSOX Red and JC-10 were purchased from Sigma-Aldrich. Hydroxyl radical (•OH) generated in the Fenton system from ferrous sulfate and H_2O_2. Singlet oxygen ($^1O_2$) was produced from the HClO-H_2O_2 system in PBS buffer. Superoxide ($O_2^{\bullet-}$) was gotten by dissolving KO_2 in DMSO. Peroxynitrite ($ONOO^-$) was obtained by mixing $NaNO_2$ (1 mM) and H_2O_2 (1 mM). Nitric oxide (NO) originated from $ONOO^-$ (1 mM). The H_2O_2 working solution was diluted immediately from a stabilized 30% solution. The ^1H and ^{13}C NMR spectra were obtained from Bruker DRX500 spectrometer in DMSO-d_6 or $CDCl_3$ at 25ºC. MALDI-TOF mass spectra were recorded using Bruker Autoflex III Smartbeam. The UV-*vis* absorption spectra of dilute solutions were recorded using a U-3900H spectrophotometer. One-photon fluorescence spectra were obtained using a HITACHI F-7100 spectrofluorometer equipped with a 450 W Xe lamp. The living cell fluorescent imaging was obtained by Nikon N-SIM E microscope and Nikon NIS-Elements system. The fluorescence lifetime measurements were performed using Edinburgh Instruments FLS1000 manufactured with pride in the United Kingdom. Refrigeration centrifuge (5810R, Thermo Fisher, USA) was used for collecting cells. Cell viability was assessed using a microplate reader (Infinite M200 Pro, Tecan, Switzerland). All measurements were performed at room temperature unless otherwise noted. Ultrapure water was used to prepare all aqueous solutions.

2. Chemical Synthesis

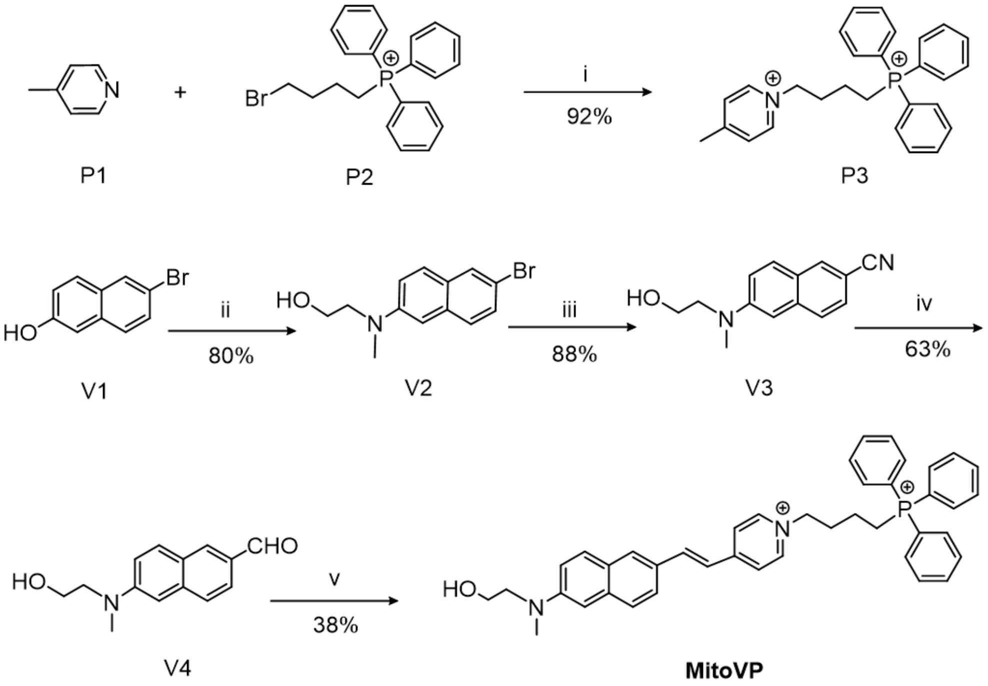

Scheme S1. The synthetic routes of viscosity fluorogenic probe (**MitoVP**). (i) DMSO, triphenylphosphine, 80°C, 10 h; (ii) 2-(Methylamino)ethan-1-ol, $Na_2S_2O_5$, microwave, 165°C, 6 h; (iii) CuCN, CH_3CN, microwave, 220°C, 4 h; (iv) Diisobutyl aluminium hydride (DIBAL), -78°C, 2 h; (v) P3, 1, 4-dimethyl-1λ^4-pyridin-2-ylium, ethanol (EtOH), 75°C, 4 h.

Synthesis of P3

These organic intermediates were synthesized according to the literature method.[1] P1 (2.3 g, 14.27 mmol) and P2 (3.5 g, 12.72 mmol) were combined in a round-bottom flask (100 mL) with DMSO (30 mL). The mixture was heated to 80°C and stirred under reflux for 12 h. After cooling to room temperature, the reaction was quenched by pouring into EA. A white precipitate formed, which was collected by filtration to afford the product (4.31 g, 90% yield).

Synthesis of V2

6-Bromo-2-naphthol (1.11 g, 5 mmol), 2-(Methylamino)ethan-1-ol (3.75 g, 50 mmol), and $Na_2S_2O_5$ (4.75 g, 25 mmol) were combined in a microwave tube (20 mL). After adding water to a specified volume, the mixture was reacted under microwave activation at 140°C for 24 h. The solution was then cooled to room temperature, resulting in the precipitation of solid material. The solid was isolated by dispersing in water and washing with DCM. The crude product was obtained by removing the solvent using a rotary evaporator, which was subsequently purified by column chromatography (V_{EA} : V_{PE} = 1 : 10) to obtain a white solid (V2, 1.12 g, 80% yield). ^{1}H NMR (500 MHz, $CDCl_3$), δppm: 7.83 (d, J = 1.8 Hz, 1H), 7.61 (d, J = 9.1 Hz, 1H), 7.51 (d, J = 8.8 Hz, 1H), 7.42 (dd, J = 8.8, 2.0 Hz, 1H), 7.21 (dd, J = 9.1, 2.6 Hz, 1H), 6.94 (d, J = 2.4 Hz, 1H), 3.88 (t, J = 5.4 Hz, 2H), 3.58 (t, J = 5.7 Hz, 2H), 3.07 (s, 3H).

Synthesis of V3.

V2 (1.12 g, 4 mmol), CuCN (2.13 g, 24 mmol), and pyridine (13 mL) were added to a microwave tube (20 mL). The reaction mixture was heated at 220°C for 3 h. Upon completion, the reaction mixture was cooled to room temperature, followed by the addition of water (30 mL). The resulting mixture was then extracted with EA, and the organic phases were combined and dried over anhydrous Na_2SO_4. The crude product was concentrated by evaporation and purified by column chromatography (V_{EA} : V_{PE} = 2 : 1), resulting in the formation of a gray solid (V3, 795 mg, 88% yield). ^{1}H NMR (500 MHz, $CDCl_3$) δppm: 8.02 (s, 1H), 7.71 (d, *J* = 9.2 Hz, 1H), 7.63 (d, *J* = 8.6 Hz, 1H), 7.45 (dd, *J* = 8.6, 1.6 Hz, 1H), 7.25 (d, *J* = 2.6 Hz, 1H), 6.93 (t, *J* = 11.8 Hz, 1H), 3.90 (q, *J* = 5.5 Hz, 2H), 3.66 (t, *J* = 5.7 Hz, 2H), 3.14 (s, 3H), 1.62 (t, *J* = 5.6 Hz, 1H).

Synthesis of V4.

V3 (678 mg, 3 mmol) and toluene (8 mL) were added into a round bottom flask (50 mL) under N_2 protection. Then, the mixture was slowly added dropwise DIBAL (1.0 M solution in hexanes; 3.6 mL, 3.6 mmol) at -78°C, and stirred for 30 min. The mixture was allowed to warm to room temperature gradually. The progress was monitored using TLC until V4 was completely formed. The reaction was then quenched with a saturated NH_4Cl aqueous solution and extracted with EA. The organic layer was dried overnight with anhydrous $MgSO_4$. The crude product was obtained by removing the solvent using a rotary evaporator. The residue was subsequently purified by column chromatography (V_{EA} : V_{PE} = 1 : 1) to obtain a yellow sheet-like crystal (V4, 653 mg, 95% yield). ^{1}H NMR (500 MHz, $CDCl_3$) δppm: 10.01 (s, 1H), 8.08 (d, *J* = 62.4 Hz, 1H), 7.82 (d, *J* = 8.9 Hz, 1H), 7.68 (dd, *J* = 25.2, 8.9 Hz, 1H), 7.53-7.36 (m, 1H), 7.24 (dd, *J* = 7.4, 2.4 Hz, 1H), 6.93 (dd, *J* = 21.1, 2.5 Hz, 1H), 3.91 (p, *J* = 5.6 Hz, 2H), 3.66 (dt, *J* = 7.9, 5.7 Hz, 2H), 3.15 (d, *J* = 8.7 Hz, 3H), 1.67 (q, *J* = 5.6 Hz, 1H). HR-MS: (m/z, ESI): Cal. For $[C_{14}H_{15}NO_2+H]^+$, m/z = 230.1103; $[M+H]^+$, found, m/z = 230.1411.

Synthesis of MitoVP.

V4 (1.0 g, 3.22 mmol), P3 (1.25 g, 3.22 mmol), and absolute ethanol (25 mL) added into a one-necked flask (100 mL). The piperidine (five drops) were added to the mixture and refluxed for 24 h. After cooling, the solution was filtered, and the solid was washed thrice with ether. Purplish red powdered product (**MitoVP**, 0.92 g, 38% yield) was collected. ^{1}H NMR (500 MHz, DMSO-d_6), δ ppm: 8.98 (dd, *J* = 18.2, 6.6 Hz, 2H), 7.99 (d, *J* = 6.3 Hz, 2H), 7.94-7.74 (m, 23H), 4.66 (t, *J* = 7.2 Hz, 2H), 4.02 (q, *J* = 7.1 Hz, 2H), 3.85-3.63 (m, 3H), 3.10-2.89 (m, 2H), 2.56-2.46 (m, 2H), 2.17-2.12 (m, 2H), 1.99 (s, 3H). ^{13}C NMR (125 MHz, DMSO-d_6), δ (ppm): 170.84, 159.37, 144.35, 144.18, 134.07, 128.63, 119.47, 119.15, 118.30, 60.24, 58.93, 58.76, 58.63, 56.47, 44.07, 31.61, 31.43, 21.26, 14.56. HR-MS: (m/z, ESI): Cal. For $[C_{42}H_{43}N_2OP/2]^{2+}$, m/z = 311.1551; $[M/2]^{2+}$, found, m/z = 311.7326.

3. Results and Discussion

3.1 Supplemental Figures

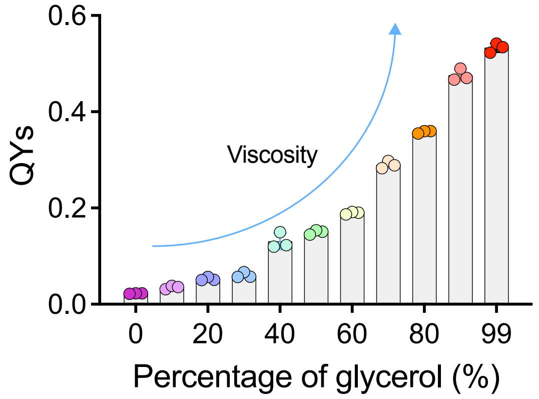

Figure S1. The percentage of fluorescence quantum yields (QYs) of **MitoVP** under increasing solution viscosity.

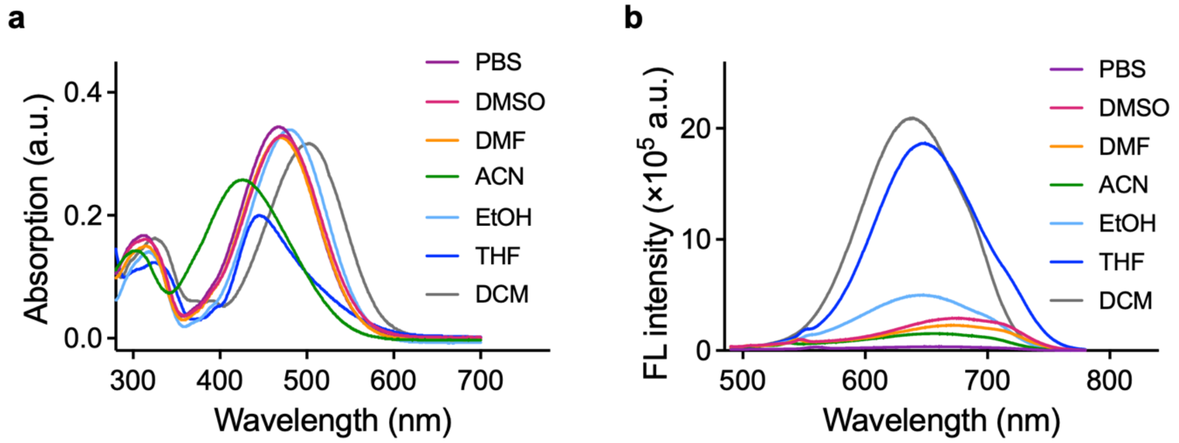

Figure S2. The linear absorption (a) and emission (b) spectra of **MitoVP** (10 μM) in solvents of varying polarity.

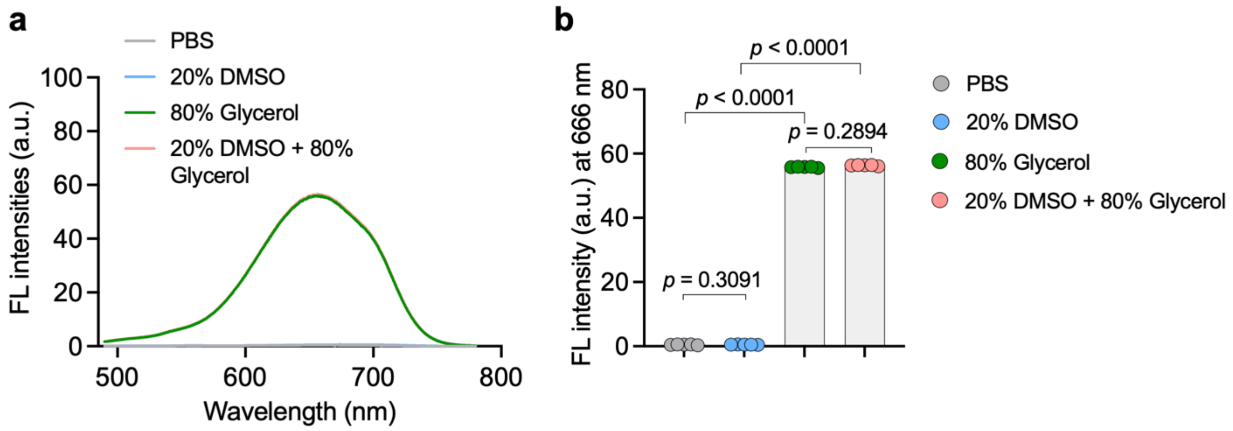

Figure S3. (a) The emission spectra and fluorescence intensity at 666 nm (b) of **MitoVP** (10 μM) after incubation with different matrix-like polarity conditions, statistical differences were calculated using a two-tailed Student's t-test.

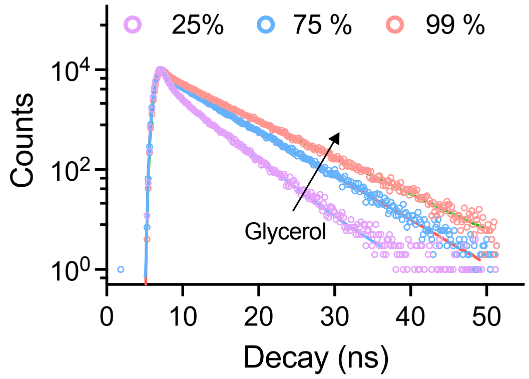

Figure S4. Fluorescence lifetimes of **MitoVP** under increasing solution viscosity (25%, 75%, 99%).

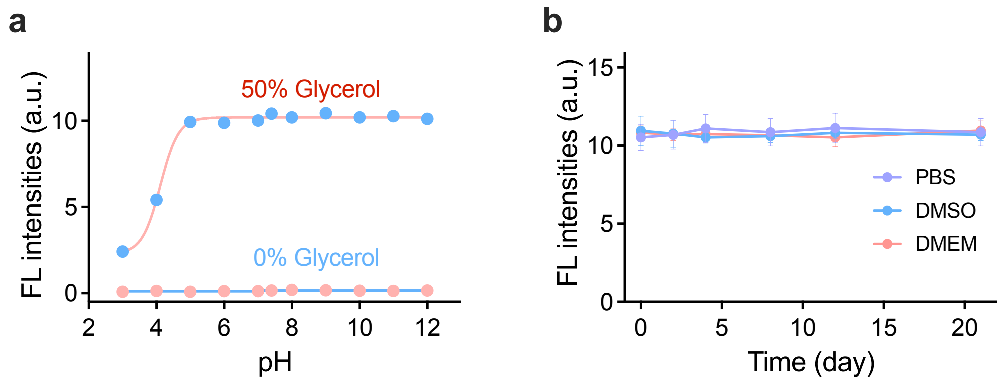

Figure S5. (a) Emission intensity changes of **MitoVP** (10 μM) at various pH values. (b) Fluorescence intensities of **MitoVP** (10 μM) over time in PBS, DMSO and DMEM, n = 3.

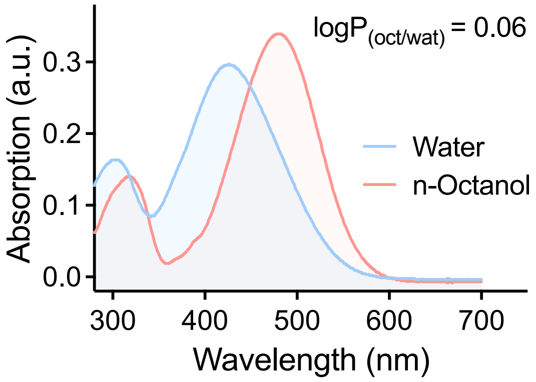

Figure S6. Absorption spectra of **MitoVP** (10 μM) in water and *n*-Octanol (containing 0.5% DMSO), with corresponding oil-water partition coefficient (log $P_{oct/wat}$ = 0.06).

Noted: Multiple factors influence the membrane permeability of substances, with the primary determinants being molecular weight, lipophilicity, and charge. The probe **MitoVP** has a relative molecular mass of 622 and an oil-water partition coefficient (log $P_{oct/wat}$) of 0.06, which falls within the acceptable range (log $P < 5$) for solubility and membrane permeability.[2] In addition, **MitoVP** is a cationic amphiphilic molecule, which facilitates its ability to penetrate the cell membrane.[3]

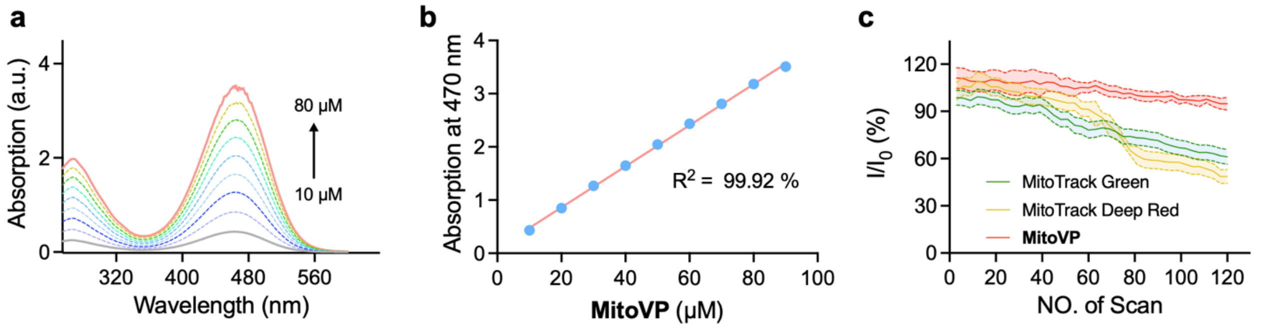

Figure S7. (a) Absorption spectra and corresponding plot (b) of intensity at various concentration of **MitoVP** in aqueous solution. (c) Loss in fluorescence of HepG2 cells treated with MitoTracker Green, MitoTracker DeepRed, and **MitoVP** by increasing the number of scans. Scanning rate: 22.4 s frame^{-1}.

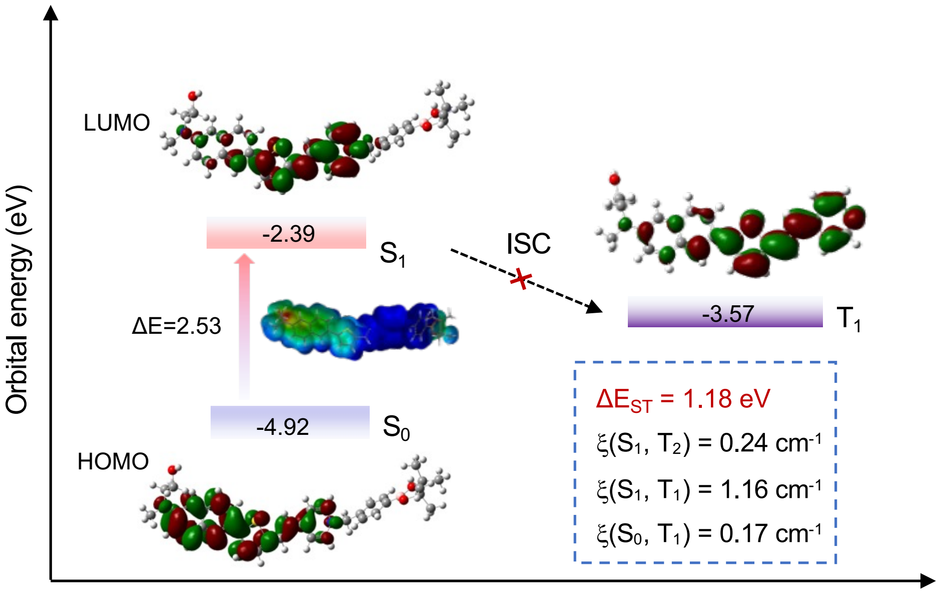

Figure S8. The frontier molecular orbitals of the **MitoVP** were calculated by density functional theory (DFT). The highest occupied molecular orbitals (HOMOs) represent the ground states, while the lowest unoccupied molecular orbitals (LUMOs) correspond to the excited states. Electrostatic potential (ESP) maps were plotted with a color scale ranging from blue (+2.0 e, positive) to red (-2.0 e, negative).

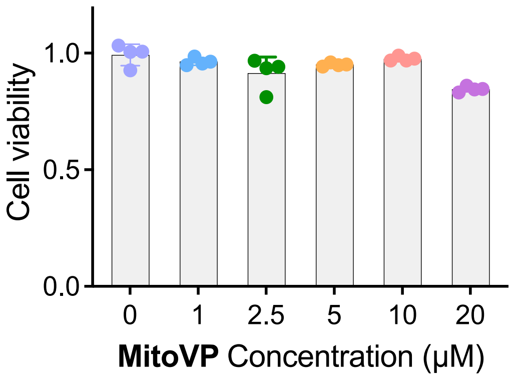

Figure S9. Cell viability of HepG2 cells treated with **MitoVP** at various concentrations (0, 1, 2.5, 5, 10, and 20 μM), n = 4.

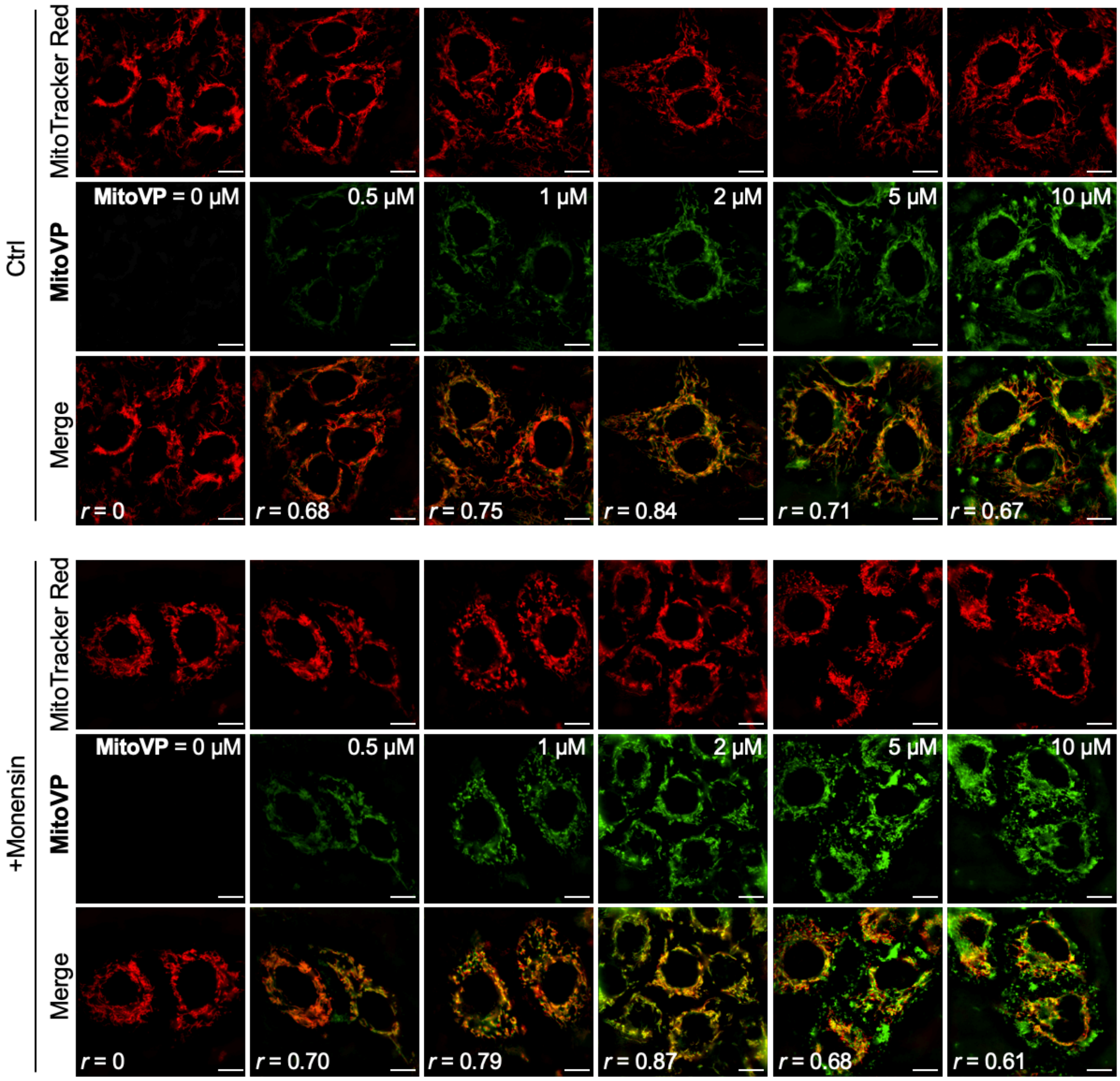

Figure S10. SIM images and Pearson's correlation coefficients (*r*) showing colocalization of MitoTracker Red (500 nM, 30 min) and MitoVP (0, 0.5, 1, 2, 5, and 10 μM, 30 min) in control and monensin-treated (10 μM, 30 min) HepG2 cells, scale bar = 10 μm.

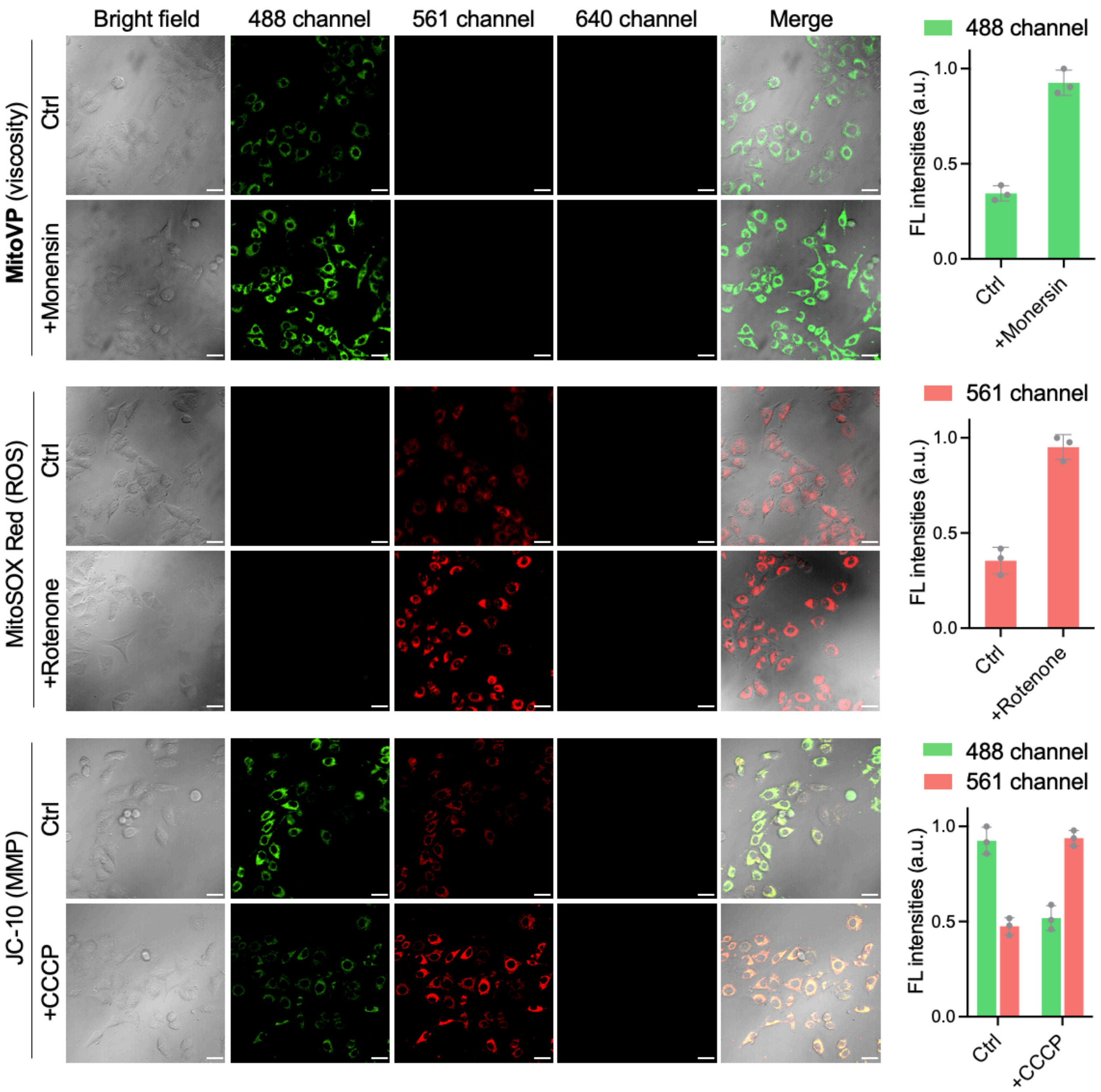

Figure S11. Single-probe fluorescence imaging to evaluate channel specificity. HepG2 cells were stained individually with **MitoVP** ($\lambda_{ex}/\lambda_{em}$ = 480/666 nm, microscope channel: $\lambda_{ex}/\lambda_{em}$ = 488/700 nm, 2 μM, 30 min), MitoSOX Red ($\lambda_{ex}/\lambda_{em}$ = 510/610 nm, microscope channel: $\lambda_{ex}/\lambda_{em}$ = 488/605 nm, 5 μM, 30 min), or JC-10 (red channel: $\lambda_{ex}/\lambda_{em}$ = 540/590 nm, microscope channel: $\lambda_{ex}/\lambda_{em}$ = 561/605 nm; green channel: $\lambda_{ex}/\lambda_{em}$ = 490/525 nm, microscope channel: $\lambda_{ex}/\lambda_{em}$ = 488/510 nm; 10 μM; 30 min), followed by confocal imaging across 488, 561, and 640 nm channels, scale bar = 50 μm.

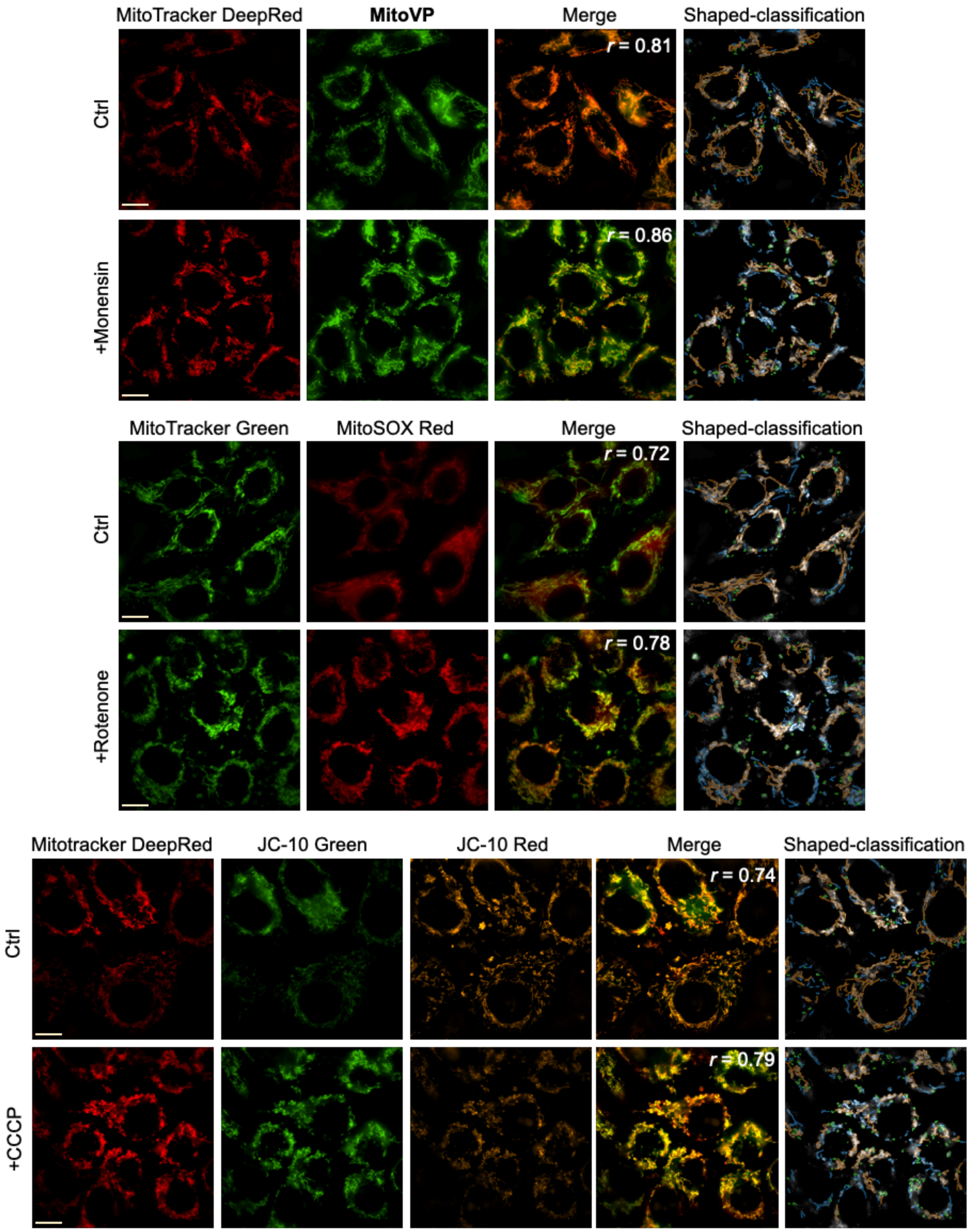

Figure S12. SIM imaging illustrating mitochondrial colocalization and corresponding shaped-classified images. HepG2 cells were treated with monensin (10 μM, 1 h), rotenone (10 μM, 4 h), or CCCP (10 μM, 4 h), followed by staining with MitoTracker Green ($\lambda_{ex}/\lambda_{em}$ = 490/516 nm, microscope channel: $\lambda_{ex}/\lambda_{em}$ = 488/510 nm) or MitoTracker DeepRed ($\lambda_{ex}/\lambda_{em}$ = 644/665 nm, microscope channel: $\lambda_{ex}/\lambda_{em}$ = 640/700 nm). Simultaneously, fluorescent probes were used to assess mitochondrial functional biomarkers: **MitoVP** ($\lambda_{ex}/\lambda_{em}$ = 480/666 nm, microscope channel: $\lambda_{ex}/\lambda_{em}$ = 488/700 nm, 2 μM, 30 min), MitoSOX Red ($\lambda_{ex}/\lambda_{em}$ = 510/610 nm, microscope channel: $\lambda_{ex}/\lambda_{em}$ = 488/605 nm, 5 μM, 30 min), JC-10 (red channel: $\lambda_{ex}/\lambda_{em}$ = 540/590 nm, microscope channel: $\lambda_{ex}/\lambda_{em}$ = 561/605 nm; green channel: $\lambda_{ex}/\lambda_{em}$ = 490/525 nm, microscope channel: $\lambda_{ex}/\lambda_{em}$ = 488/510 nm; 10 μM; 30 min) for viscosity, ROS level, and MMP depolarization, respectively, scale bar = 10 μm. Pearson's correlation coefficients (*r*) indicate colocalization levels, **MitoVP** displaying the highest colocalization with MitoTracker ($r > 0.8$).

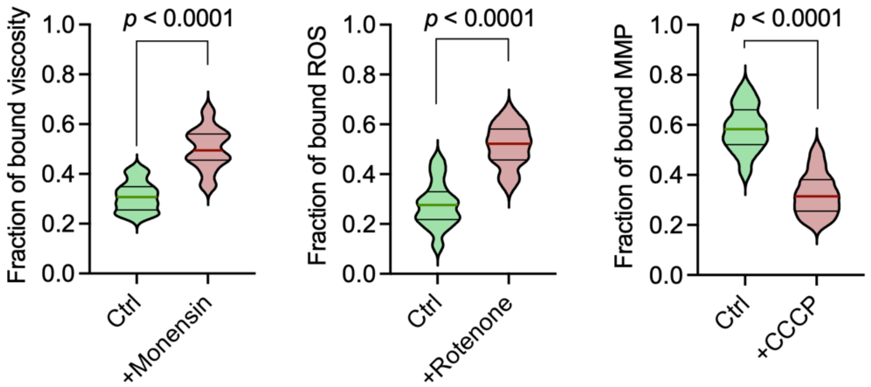

Figure S13. Violin plots comparing the fraction of bound biomarkers between control and drug-treated groups (+monensin, 10 μM, 1 h; +rotenone, 10 μM, 4 h; +CCCP, 10 μM, 4 h), statistical differences were calculated using a two-tailed Student's t-test.

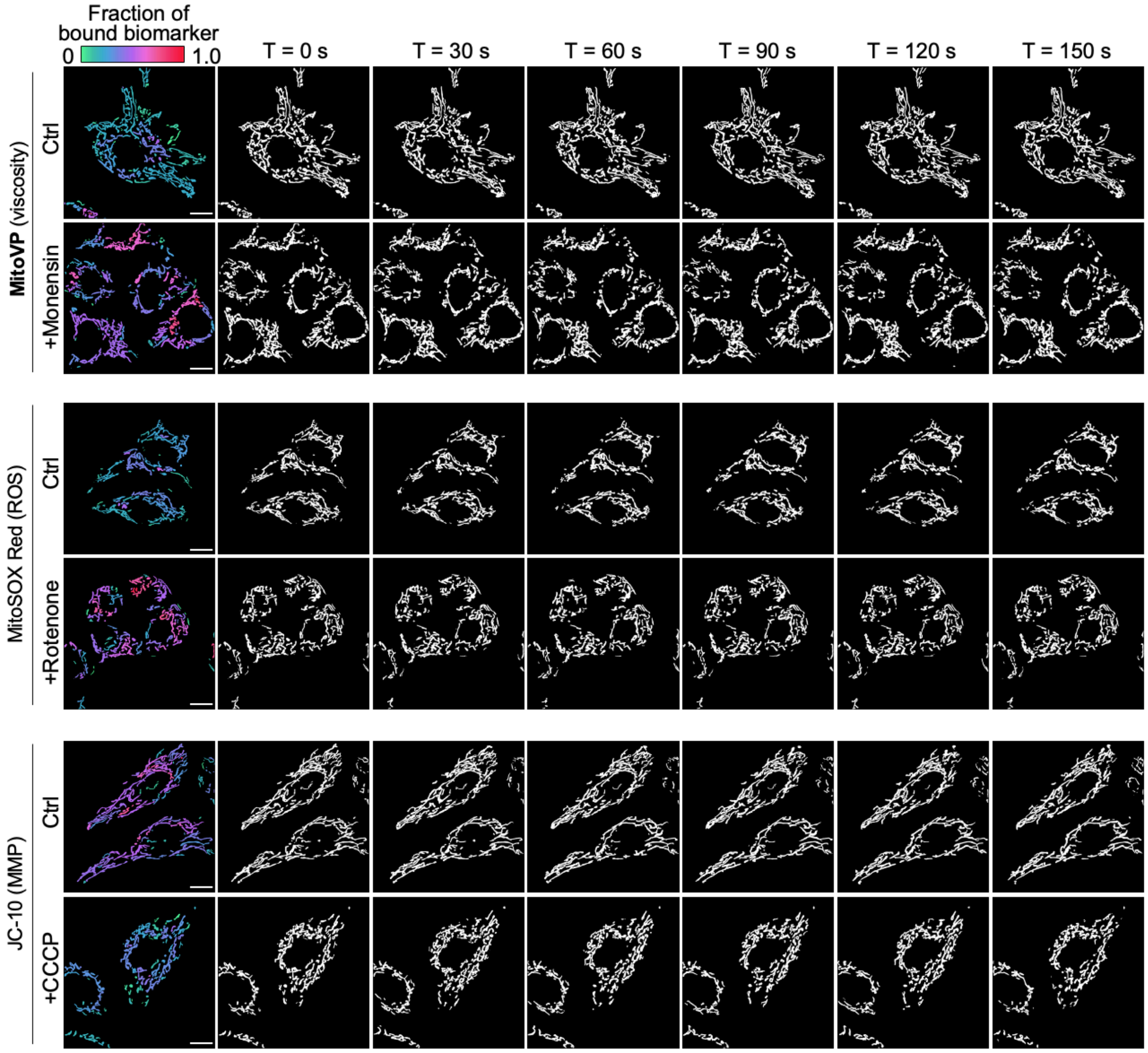

Figure S14. Time-lapse imaging of mitochondria labeled with **MitoVP**, MitoSOX Red, and JC-10 in control and drug-treated HepG2 cells (monensin, rotenone, and CCCP, respectively). Pseudo-color images (T = 0 s) show the spatial distribution of bound biomarker fractions. Sequential segmentation masks (T = 0, 30, 60, 90, 120, and 150 s) reveal mitochondrial dynamics over time, scale bar = 10 μm.

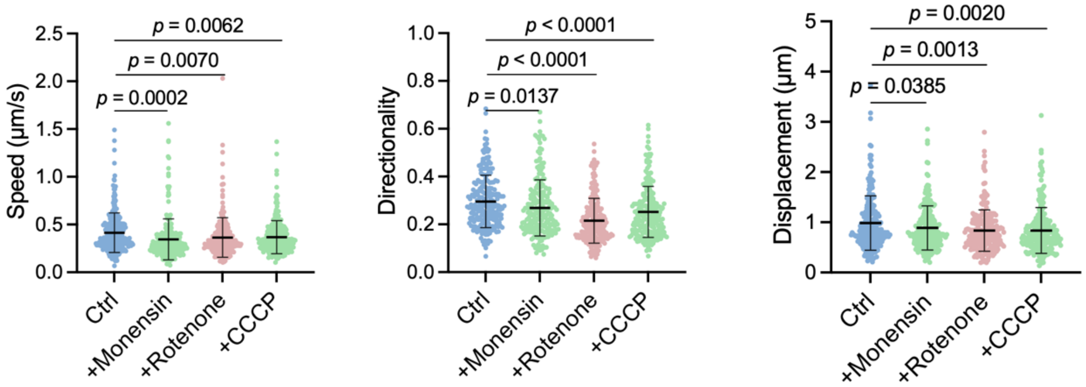

Figure S15. Quantitative comparison of mitochondrial dynamic parameters (motility speed, directionality, and displacement) between control and drug-treated HepG2 cells (monensin, rotenone, and CCCP, respectively), calculated using the Mitometer plugin,[4] n = 225 mitochondria, statistical differences were calculated using a one-way ANOVA followed by Dunnett's multiple comparison test.

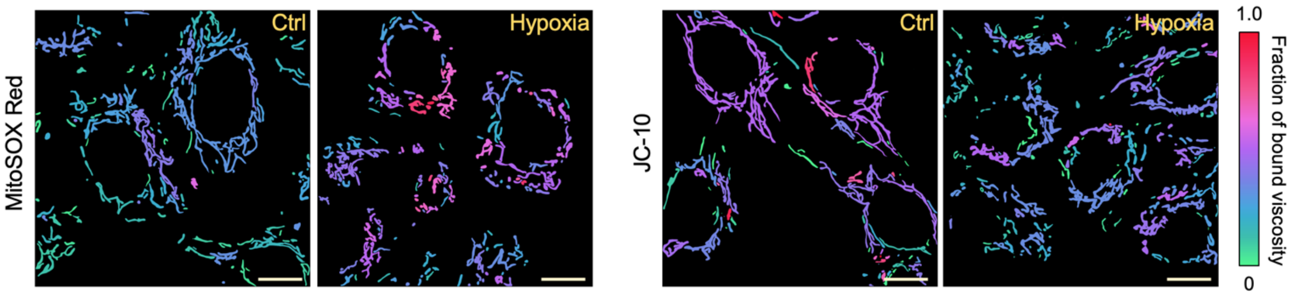

Figure S16. Representative merged pseudo-color in control and hypoxia-treated HepG2 cells. Hypoxic condition was performed by culturing cells under 1% O_2 for 6 h, followed by staining with MitoSOX Red (5 µM, 30 min) or JC-10 (10 µM, 30 min) prior to imaging, scale bar = 10 µm.

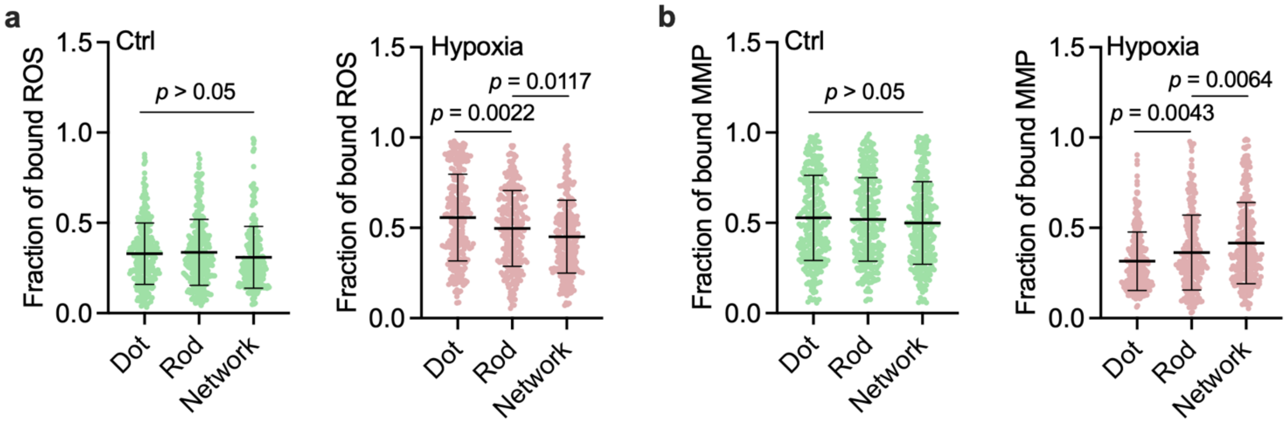

Figure S17. Comparison of fraction of bound ROS (a) and MMP (b) among mitochondria classified as dot, rod, or network under control and hypoxic conditions, n = 429 mitochondria, statistical differences were calculated using a two-tailed Student's t-test.

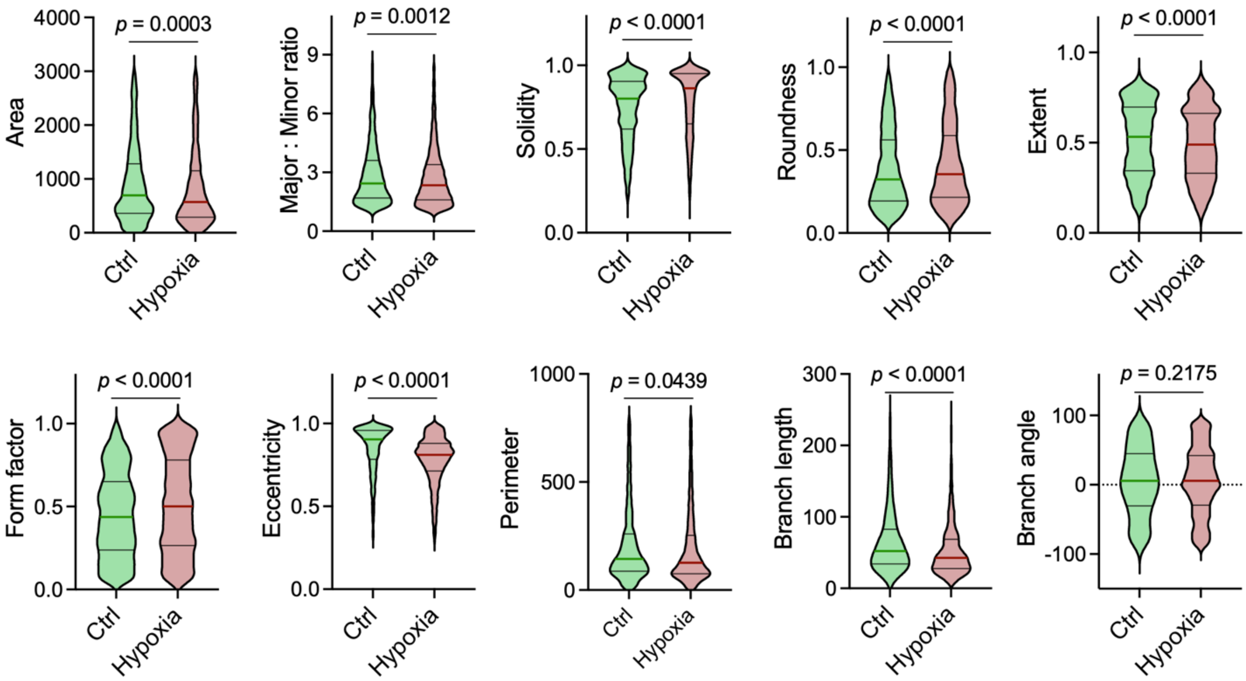

Figure S18. Comparison of multiple mitochondrial morphological parameters between control and hypoxic cells, calculated from mask-based feature extraction, statistical differences were calculated using a two-tailed Student's t-test.

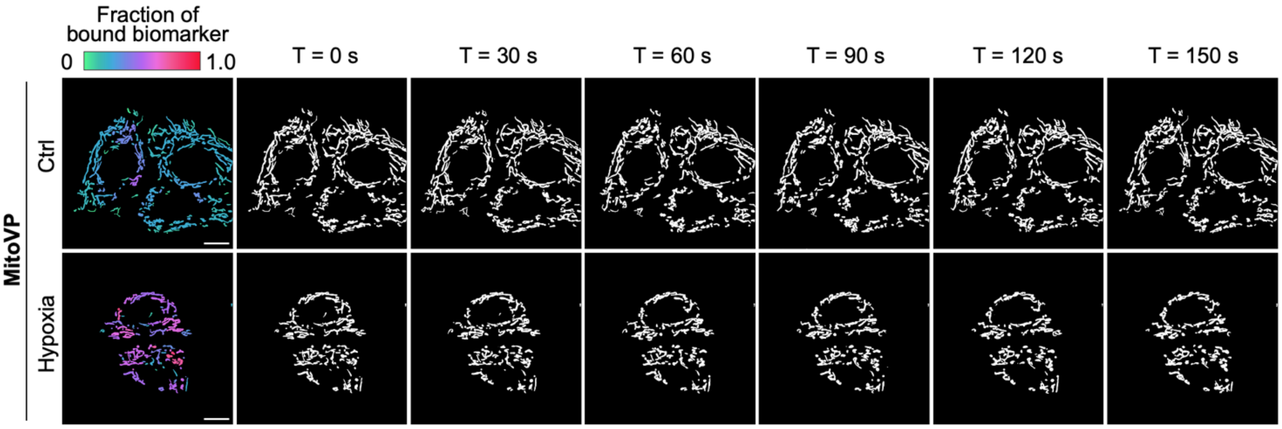

Figure S19. Time-lapse imaging of mitochondria labeled with **MitoVP** in control and hypoxia-treated HepG2 cells. Pseudo-color images (T = 0 s) and sequential segmentation masks (T = 0, 30, 60, 90, 120, and 150 s) reveal mitochondrial dynamics over time, scale bar = 10 μm.

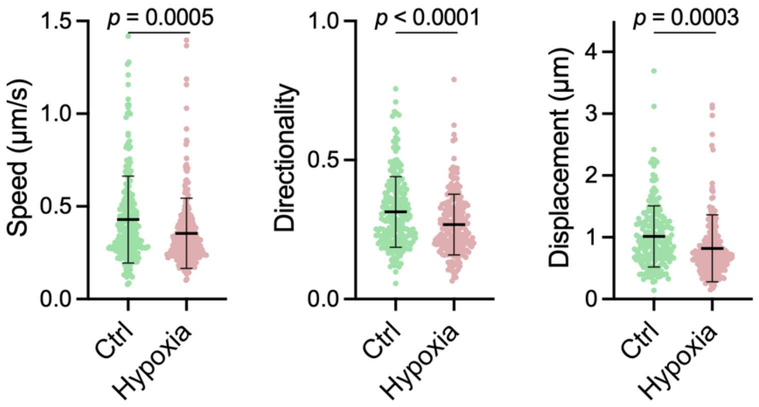

Figure S20. Quantitative comparison of mitochondrial dynamic parameters between control and hypoxia-treated HepG2 cells, n = 225 mitochondria, statistical differences were calculated using a two-tailed Student's t-test.

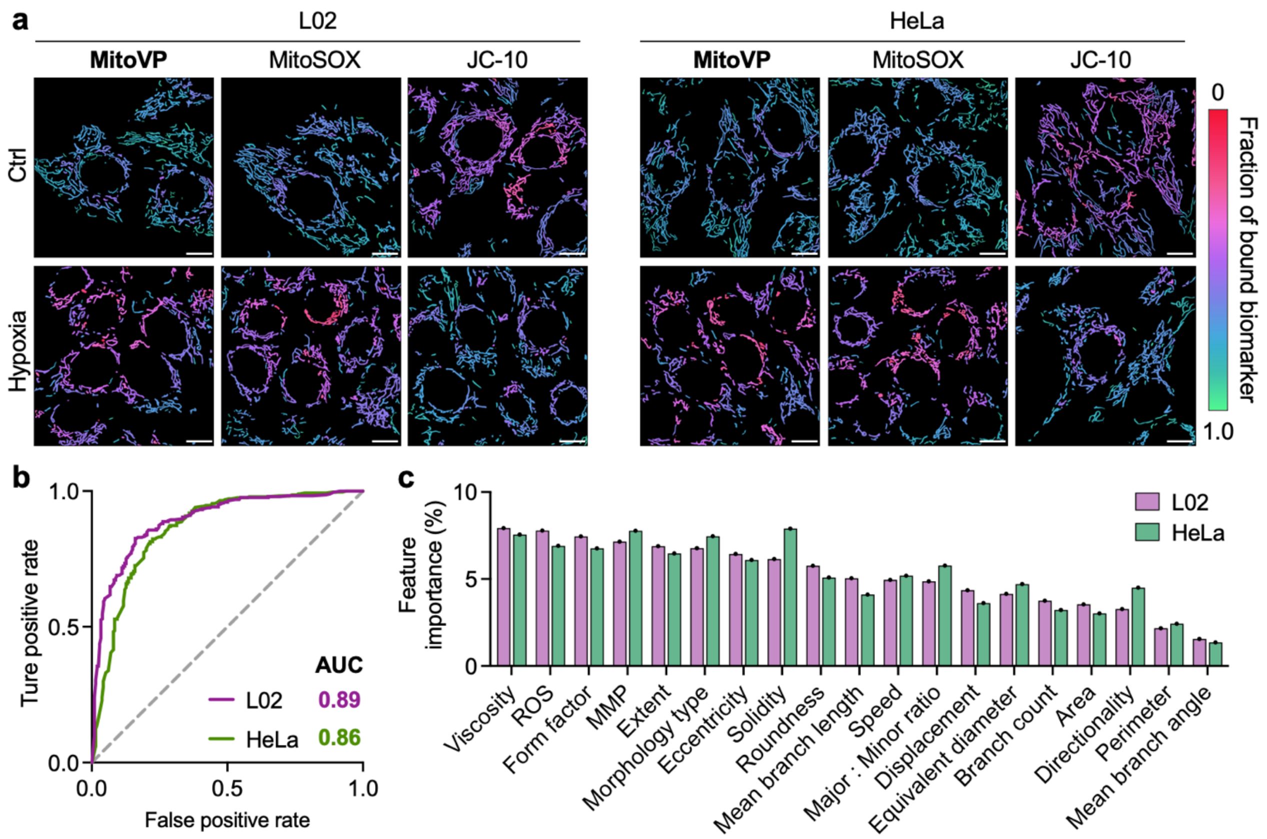

Figure S21. (a) Representative merged pseudo-color images of control and hypoxia-treated L02 and HeLa cells, staining with **MitoVP** (2 μM, 30 min), MitoSOX Red (5 μM, 30 min) or JC-10 (10 μM, 30 min) prior to imaging, scale bar = 10 μm. (b) ROC curves and AUC values evaluating the performance of the random forest classifier in L02 and HeLa tasks. (c) Feature importance identifying the relative contributions of morphological, dynamic, and biomarker features for mitochondrial classification under control and hypoxic conditions.

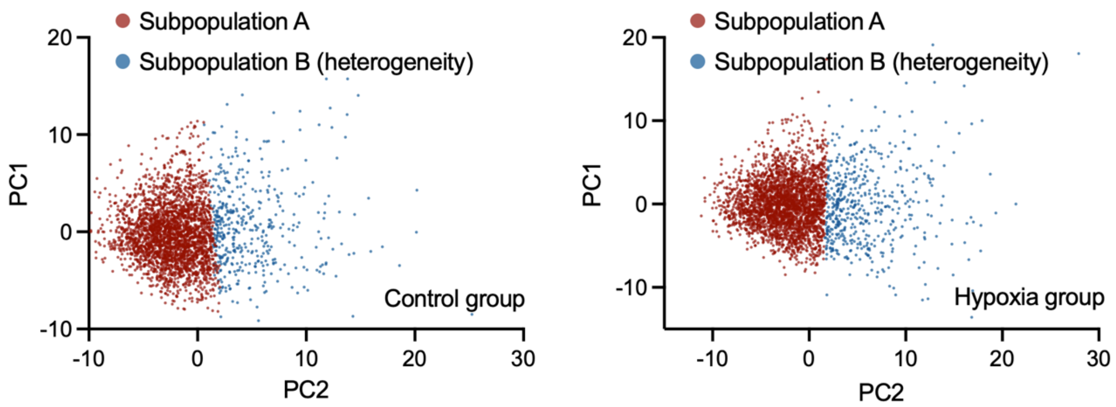

Figure S22. Principal component analysis (PCA) visualization of control (n = 3,197 mitochondria; subpopulation A = 2,749; subpopulation B = 448; heterogeneity proportion = 15.3%) and hypoxia group (n = 3,295 mitochondria; subpopulation A = 2,682; subpopulation B = 613; heterogeneity proportion = 18.6%) mitochondrial heterogeneity identified by K-means (*k* = 2) clustering.

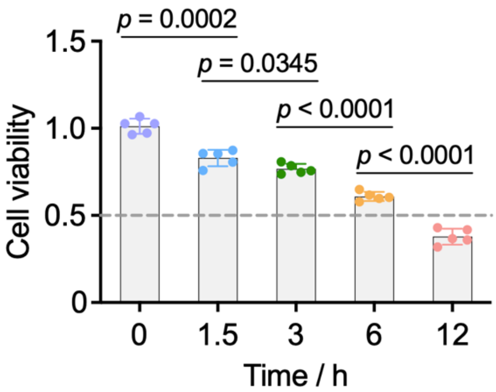

Figure S23. Cell viability of hypoxia-treated HepG2 cells assessed at 0 (control), 1.5 (mild), 3 (moderate), 6 (severe), and 12 h (cell viability < 50%, imaging-incompatible) time points, n = 5, statistical differences were calculated using a two-tailed Student's t-test.

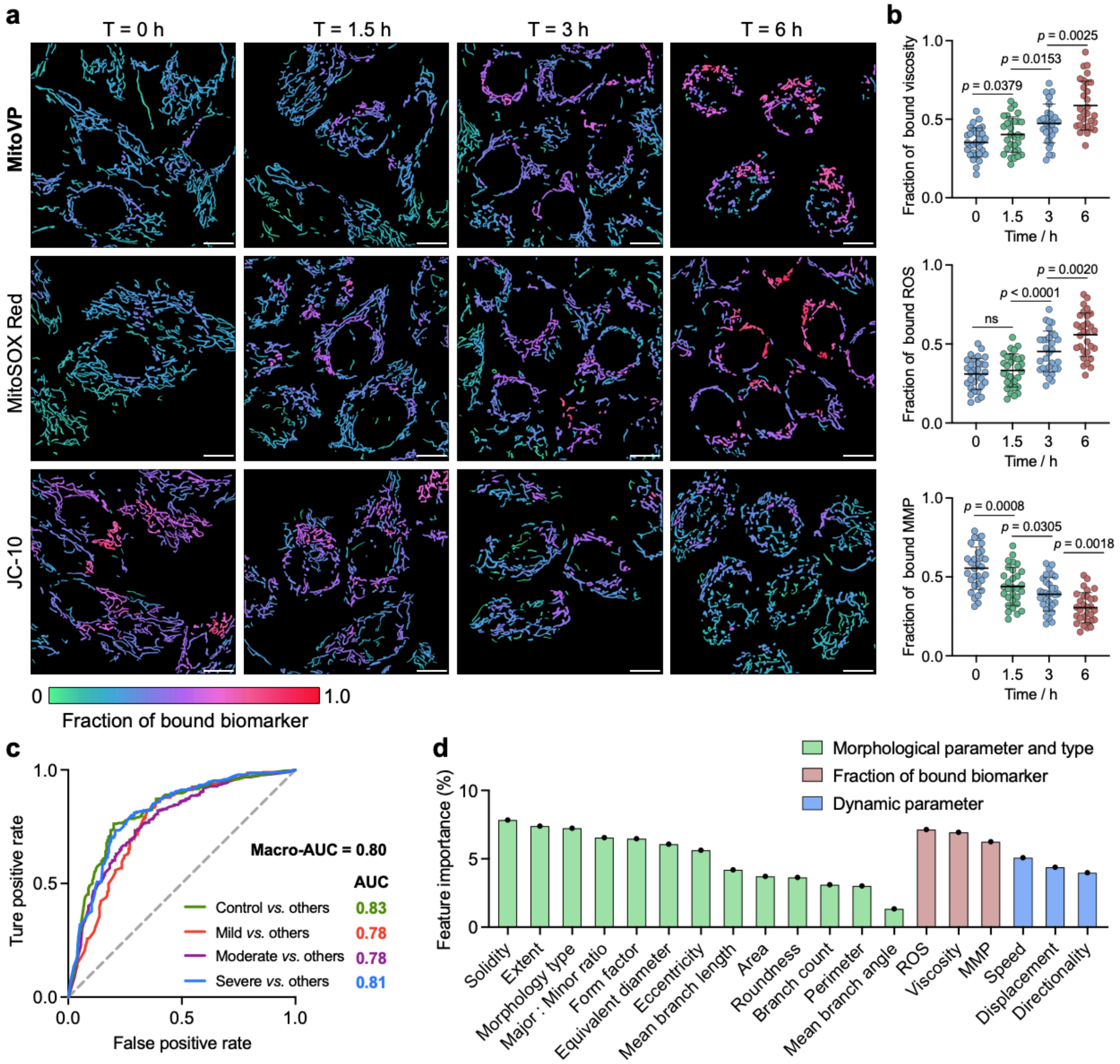

Figure S24. (a) Representative merged pseudo-color and corresponding fractions of bound biomarkers (b) in hypoxia-treated HepG2 cells at 0 (control), 1.5 (mild), 3 (moderate), and 6 h (severe), staining with **MitoVP** (2 μM, 30 min), MitoSOX Red (5 μM, 30 min) or JC-10 (10 μM, 30 min) prior to imaging, scale bar = 10 μm, n = 30 images. (c) ROC curves for multi-label classification performance based on a one-vs-rest strategy. The macro-averaged AUC across all classes is 0.80, indicating overall discriminative performance of the model. (d) Feature importance ranking in the multi-label prediction model. Statistical differences were calculated using a two-tailed Student's t-test (b).

3.2 Supplemental Tables

TableS1. Photophysical properties of the **MitoVP** in different solvents.

Solvents	λ_{ex}[a]	λ_{em}[b]	ΔV[c]	$Log\varepsilon_{max}$	Φ[d]	Brightness[e]
PBS	467	675	208	4.64	0.0019	82.94
DMSO	471	674	203	4.59	0.0105	408.50
DMF	469	668	199	4.59	0.0024	93.37
ACN	433	644	211	4.36	0.0016	36.65
EtOH	480	646	166	4.64	0.0119	519.45
THF	442	646	204	4.28	0.2064	3932.87
DCM	498	637	139	4.52	0.2148	7112.70

Noted: [a]Peak position of the longest absorption band in nm. [b]Peak position of emission, excited at the absorption maximum in nm. [c]Stokes' shift in nm. [d]Quantum yields determined by using rhodamine (RhB) as standard. [e]Brightness(εΦ) is proportional to the product of the extinction coefficient (ε, at the relevant excitation wavelength, 10^4)

Table S2. Calculated linear absorption properties (nm), excitation energy (eV), oscillator strengths (*f*) and major contribution for **MitoVP**.

Cmpds	λ (nm)	ΔE (eV)	*f*	Composition	Character
**MitoVP**	490.19	2.5293	0.048	166→167 (H→L) (0.69)	TICT

Table S3. Optical parameters and microscope channel settings for probes/dyes used in live-cells imaging.

		$\lambda_{ex}/\lambda_{em}$: nm	Microscope channel setting ($\lambda_{ex}/\lambda_{em}$: nm)
Probes	**MitoVP**	480/666	488/700
	MitoSOX	510/610	488/605
	JC-10	Red: 540/590<br>Green: 490/525	561/605<br>488/510
Dyes	MitoTracker Green	490/516	488/510
	MitoTracker Red	579/599	561/605
	MitoTracker DeepRed	644/665	640/700

Table S4. Sample distribution of normoxic and hypoxic mitochondria in the training-test set.

Label	Dataset	Number	Sum (10,000)
Normoxic	Training set	3,473	4,962
	Test set	1,489	
hypoxic	Training set	3,526	5,038
	Test set	1,512	

Table S5. AUC values evaluating the performance of the random forest classifier using various feature sets.

Feature sets	AUC
Biomarker fractions	0.62
Morphological type labels	0.57
Morphology parameters	0.68
Dynamic parameters	0.60
Morphology parameters + Morphological type labels	0.76
Morphology parameters + Viscosity fraction	0.75
Morphology parameters + ROS fraction	0.72
Morphology parameters + MMP fraction	0.73
Morphology parameters + Biomarker fractions	0.80
Morphology parameters + Dynamic parameters	0.78
Merge	0.87

Table S6. AUC scores from 5-fold cross-validation and repeated training with different random seeds.

Validation strategy	Details	AUC	Average
5-Fold Cross-Validation: Fold 1	Validation set: 1-2000, Seed = 42	0.865	
5-Fold Cross-Validation: Fold 2	Validation set: 2001-4000, Seed = 42	0.874	
5-Fold Cross-Validation: Fold 3	Validation set: 4001-6000, Seed = 42	0.862	0.869 ± 0.008
5-Fold Cross-Validation: Fold 4	Validation set: 6001-8000, Seed = 42	0.881	
5-Fold Cross-Validation: Fold 5	Validation set: 8001-10000, Seed = 42	0.863	
Random Seed 1	Seed = 10	0.877	
Random Seed 2	Seed = 20	0.860	
Random Seed 3	Seed = 30	0.875	0.874 ± 0.010
Random Seed 4	Seed = 40	0.871	
Random Seed 5	Seed = 50	0.886	

Table S7. Quantification of feature contributions to mitochondrial subpopulation heterogeneity under control and hypoxic conditions *via* K-means clustering.

Feature	Control		Hypoxia	
	p value	Feature importance	*p* value	Feature importance
Area	0.0813	0.0031	0.0302	0.0106
Eccentricity	0.0144	0.0042	0.0255	0.0088
Equivalent diameter	0.0053	0.0124	0.0324	0.0132
Extent	< 0.0001	0.0387	0.0003	0.0279
Major : Minor ratio	0.0092	0.0117	0.0105	0.0135
Perimeter	0.1874	0.0005	0.0418	0.0058
Solidity	< 0.0001	0.0402	< 0.0001	0.0347
Form factor	0.0083	0.0318	< 0.0001	0.0426
Roundness	0.0003	0.0279	0.0051	0.0205
Branch count	0.0582	0.0072	0.0719	0.0006
Mean branch length	0.0298	0.0117	0.0370	0.0076
Mean branch angle	0.4782	-0.0093	0.3119	0.0003
Speed	0.0233	0.0165	0.0078	0.0105
Directionality	0.0094	0.0113	0.0266	0.0093
Displacement	0.0105	0.0089	0.0090	0.0122

Note: Statistical differences (*p* value) were calculated using a two-tailed Student's t-test, subpopulation A *vs.* subpopulation B. Feature importance scores = silhouette score with all features − silhouette score after removing the feature. Higher values indicate greater contribution of the feature to clustering quality.

4. Characterization Spectra

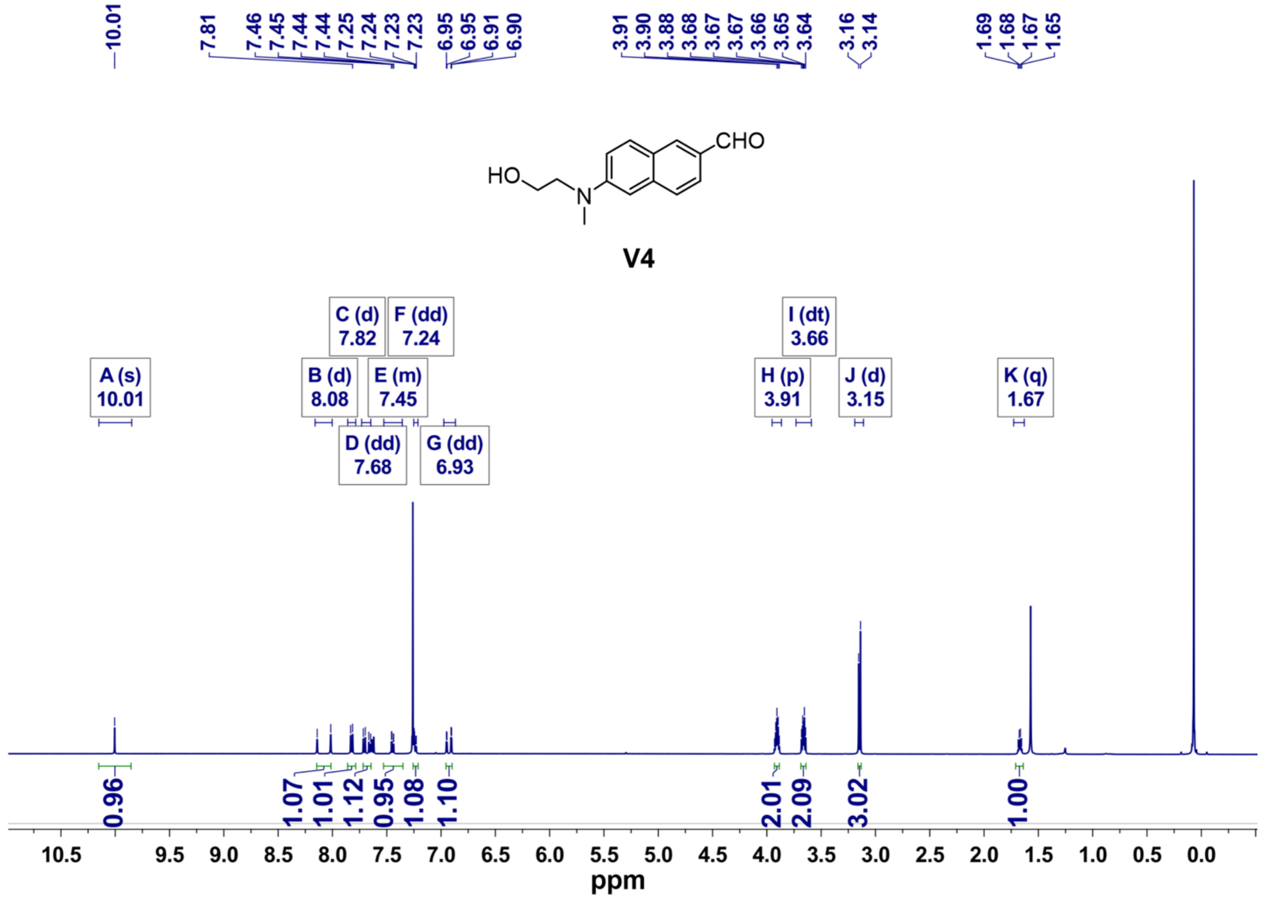

^{1}H NMR spectrum of V4 (500 MHz, 298 K, $CDCl_3$)

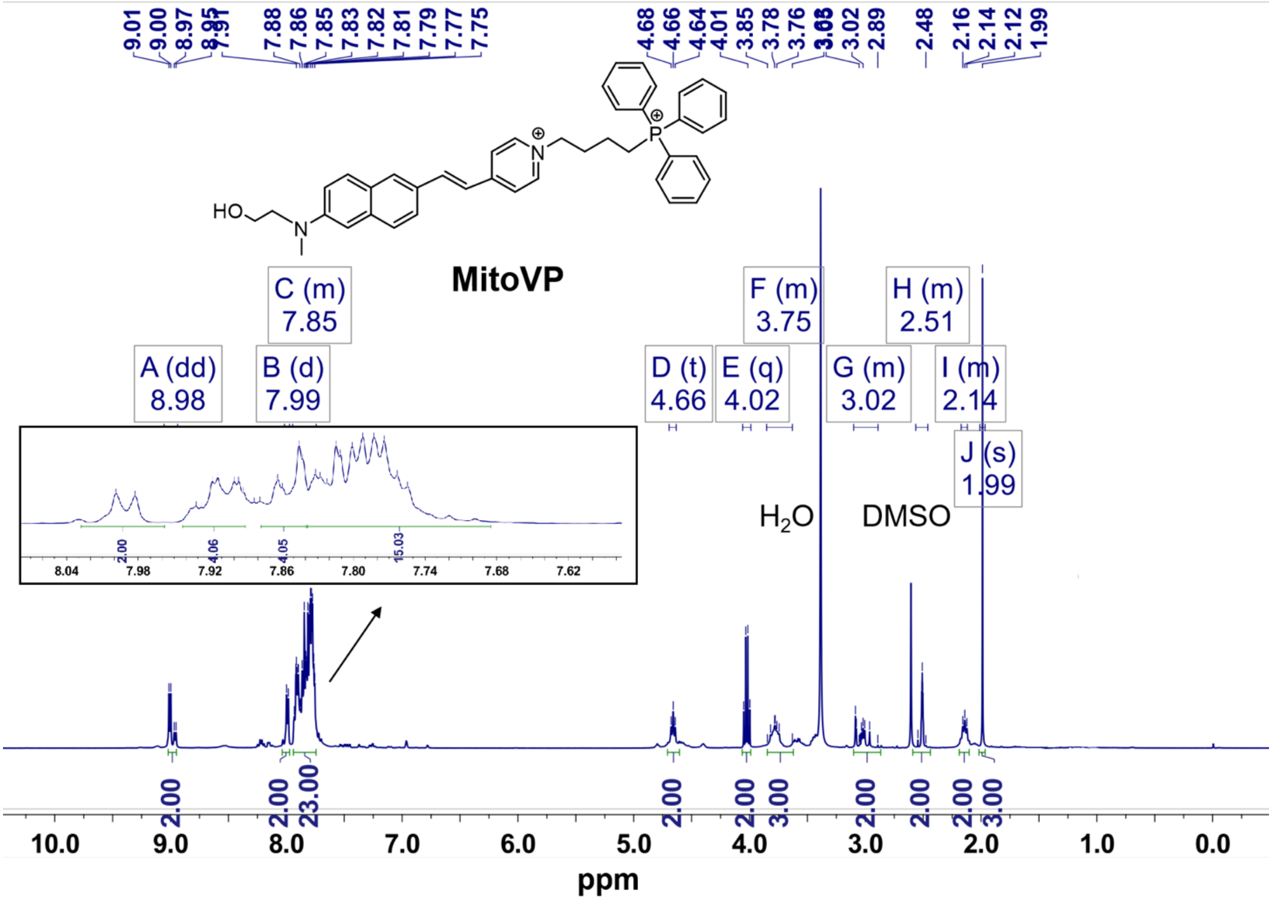

^{1}H NMR spectrum of **MitoVP** (500 MHz, 298 K, DMSO-d_6)

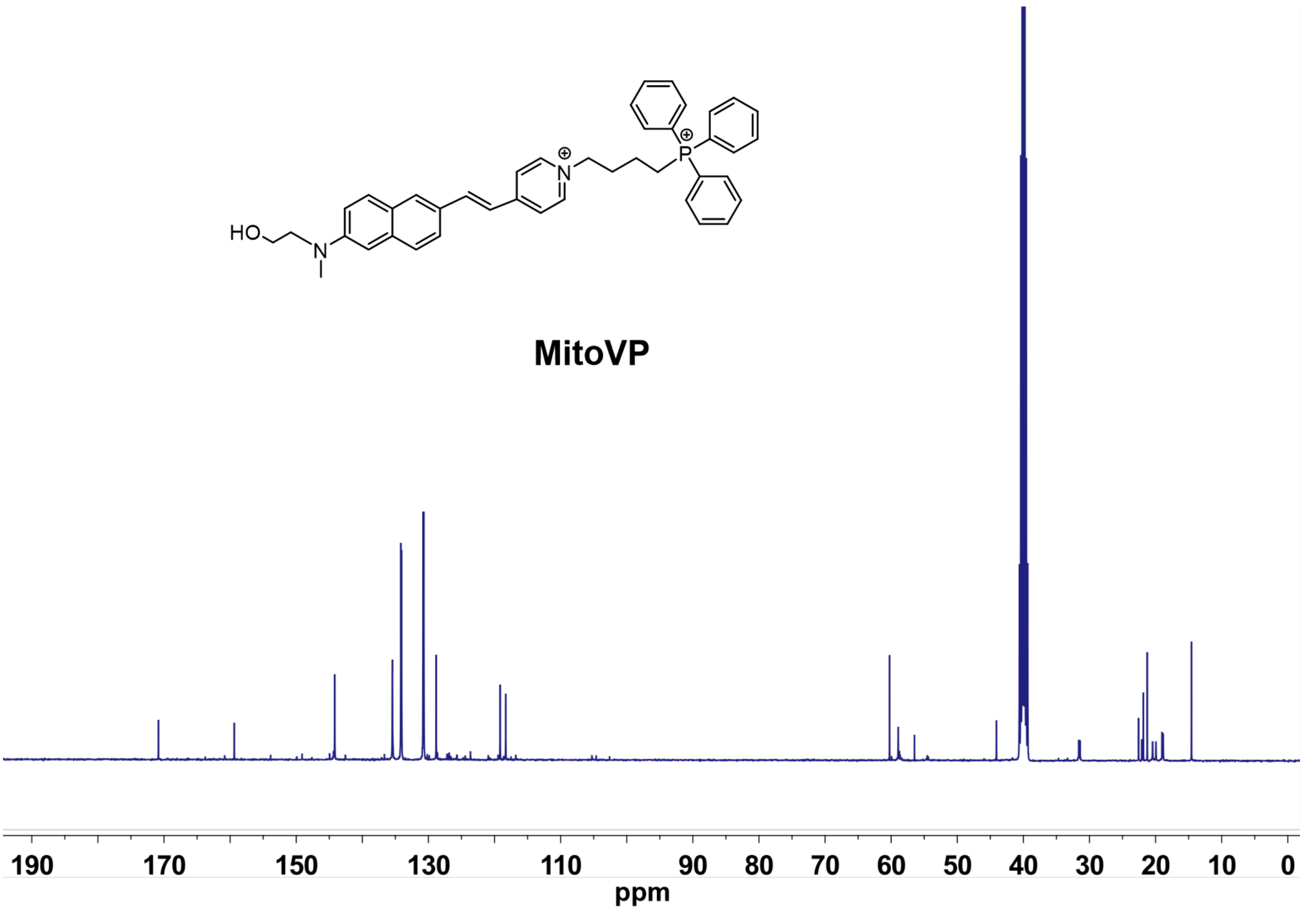

^{13}C NMR spectrum of **MitoVP** (125 MHz, 298 K, DMSO-d_6)

5. Commands for Theoretical Calculation of MitoVP

```
%nproc=6
%chk=1.chk
%mem=6000MB
# opt b3lyp/6-31g scf=xqc

0 1

 C     2.86995786    3.57968549   -4.91690753
 H     3.46614991    4.42673024   -4.64879850
 C     1.84251479    3.73058650   -5.78765454
 H     1.62570982    4.69148000   -6.20544723
 C     1.06990303    2.62249504   -6.13383895
 C     1.38521543    1.38366782   -5.57560172
 H     0.81287424    0.51586084   -5.82901563
 C     2.42270213    1.28947275   -4.70822782
 H     2.66764666    0.34187153   -4.27570042
 C    -0.11010639    2.76520015   -7.11322240
 H    -0.69331594    1.90778450   -7.37703683
 C    -0.41051299    3.97782227   -7.63925930
 H     0.17686532    4.83364572   -7.37949472
 N     3.14645079    2.37600504   -4.38590674
 C     4.25948712    2.24628480   -3.43440619
 H     5.15961150    2.01692252   -3.96536551
 H     4.38348606    3.16724223   -2.90372165
 C    -1.59764872    4.12548751   -8.60929862
 C    -2.36538297    3.00475425   -8.95445559
 C    -2.99507332    5.51570596  -10.02405380
 C    -3.44833672    3.14095848   -9.83394698
 H    -2.12501141    2.04528765   -8.54644508
 C    -3.76375678    4.39784387  -10.36755450
 H    -4.03427617    2.28540980  -10.09775222
 C    -4.84952099    4.53575260  -11.24516510
 C    -5.16286660    5.75061867  -11.76612649
 H    -5.43451044    3.67855650  -11.50562994
 C    -7.52290409    6.16713585  -11.91126509
 H    -8.35390812    6.26144937  -12.57865253
 H    -7.70640962    5.36782940  -11.22392004
 H    -7.39500515    7.08098902  -11.36954365
 C    -6.05213010    6.97830056  -13.62687816
 H    -5.16514394    6.76949608  -14.18771032
 H    -5.92464197    7.89216250  -13.08500953
 C    -7.24728043    7.11425924  -14.58861754
 H    -8.13431400    7.32316620  -14.02790138
 H    -7.37469601    6.20046760  -15.13044554
 O    -7.00120529    8.18254669  -15.50689330
 H    -7.74619891    8.26713979  -16.10647393
 N    -6.30473883    5.88009522  -12.68280759
 C    -4.40378082    6.88309250  -11.43511516
 H    -4.65636450    7.83643352  -11.85017953
 C    -3.31601365    6.76591549  -10.56183138
 H    -2.73435625    7.62720077  -10.30741419
 C    -1.91126564    5.38144391   -9.14639432
 H    -1.32396151    6.23705369   -8.88596649
 C     3.94857637    1.11640501   -2.43519558
 H     3.82816842    0.19453157   -2.96504313
 H     3.04598778    1.34586628   -1.90851339
 C     5.10780245    0.98391138   -1.42984558
 H     6.01153100    0.75282660   -1.95410250
 H     5.22829441    1.90649814   -0.90123444
 C     4.79056814   -0.14333064   -0.42941524
 H     4.67329398   -1.06628353   -0.95784527
 H     3.88474340    0.08732513    0.09125436
 C     6.35223685    1.27303466    1.66500300
```

C	7.21432394	2.25900968	1.16715582
C	5.64382073	1.49637811	2.85341219
C	7.36856181	3.46856257	1.85758758
H	7.75499179	2.08819899	0.25973734
C	5.79818803	2.70591543	3.54418137
H	4.98526902	0.74348148	3.23338548
C	6.66077094	3.69197462	3.04628667
H	8.02676686	4.22145373	1.47711328
H	5.25765431	2.87662015	4.45167720
H	6.77888644	4.61537349	3.57387811
C	5.77268321	-1.62809932	1.94931475
C	6.81131523	-2.36500613	2.53436371
C	4.44021944	-1.91559618	2.27425460
C	6.51725727	-3.38924247	3.44453589
H	7.82884510	-2.14562944	2.28617206
C	4.14607983	-2.93978969	3.18429448
H	3.64737585	-1.35298290	1.82738922
C	5.18458849	-3.67661758	3.76950493
H	7.31019242	-3.95179302	3.89139600
H	3.12857881	-3.15911010	3.43228526
H	4.96002822	-4.45869088	4.46446682
C	7.69678688	-0.69061271	-0.11375225
C	7.65595240	-1.42062967	-1.30920588
C	8.92685551	-0.26279424	0.40408356
C	8.84507824	-1.72334071	-1.98638663
H	6.71704329	-1.74697608	-1.70506900
C	10.11593993	-0.56547338	-0.27286367
H	8.95813677	0.29508980	1.31660031
C	10.07508436	-1.29597361	-1.46809160
H	8.81389447	-2.28086151	-2.89934208
H	11.05501206	-0.23878797	0.12285440
H	10.98294628	-1.52736703	-1.98494081
P	6.15330405	-0.29742014	0.76734497

```
--link1--
%nproc=6
%chk=1.chk
%mem=6000MB
# td=(singlets,nstates=50,root=1) b3lyp/6-31g(d) scf=xqc guess=read geom=allcheck
```

6. References

[1] B. Fang, L. M. Wang, H. Q. Li, J. X. Zhang, Y. Ding, P. P. Li, B. Peng, H. Bai, L. Li, “Deciphering the role of mtH_2O_2 in hepatic ischemia-reperfusion injury mechanisms with low-background super-resolution fluorogenic probe” *Sci. China Chem.* **2025**, *68*, 297-307.

[2] L. Li, H. Y. Sun, “Next generation of small-molecule fluorogenic probes for bioimaging” *Biochemistry* **2020**, *59*, 216-217.

[3] M. Z. Zhang, R. N. Su, Q. Zhang, L. Hu, X. H. Tian, Y. Chen, H. P. Zhou, J. Y. Wu, Y. P. Tian, “Ultra-bright intercellular lipids pseudo Di-BODIPY probe with low molecular weight, high quantum yield and large two-photon action cross-sections” *Sens. Actuators B Chem.* **2018**, *261*, 161-168.

[4] A. E. Y. T. Lefebvre, D. Ma, K. Kessenbrock, D. A. Lawson, M. A. Digman, “Automated segmentation and tracking of mitochondria in live-cell time-lapse images” *Nat. Methods* **2021**, *18*, 1091-1102.
